# Molecular and Cellular Mechanisms of Perineural Invasion in Oral Squamous Cell Carcinoma: Potential Targets for Therapeutic Intervention

**DOI:** 10.3390/cancers13236011

**Published:** 2021-11-29

**Authors:** Carly I. Misztal, Carlos Green, Christine Mei, Rita Bhatia, Jaylou M. Velez Torres, Brandon Kamrava, Seo Moon, Elizabeth Nicolli, Donald Weed, Zoukaa Sargi, Christine T. Dinh

**Affiliations:** 1 Department of Otolaryngology, University of Miami Miller School of Medicine, Miami, FL 33136, USA; cmisztal@med.miami.edu (C.I.M.); carlos.green@jhsmiami.org (C.G.); cmei@med.miami.edu (C.M.); brandon.kamrava@jhsmiami.org (B.K.); exn164@med.miami.edu (E.N.); dweed@med.miami.edu (D.W.); zsargi@med.miami.edu (Z.S.); 2 Department of Radiology, University of Miami Miller School of Medicine, Miami, FL 33136, USA; rbhatia@med.miami.edu; 3 Department of Pathology, University of Miami Miller School of Medicine, Miami, FL 33136, USA; jveleztorres@med.miami.edu; 4 Department of Otolaryngology, Kaiser Permanente, Oakland, CA 94611, USA; seo.y.moon@kp.org

**Keywords:** perineural invasion, PNI, oral cavity, squamous cell carcinoma, radiation, chemotherapy, neurotrophin, Schwann cells, nerve

## Abstract

**Simple Summary:**

Squamous cell carcinoma is the most common type of oral cavity cancer. It can spread along and invade nerves in a process called perineural invasion. Perineural invasion can increase the chances of tumor recurrence and reduce survival in patients with oral cancer. Understanding how oral cancer interacts with nerves to facilitate perineural invasion is an important area of research. Targeting key events that contribute to perineural invasion in oral cavity cancer may reduce tumor recurrence and improve survival. In this review, we describe the impact of perineural invasion in oral cancer and the mechanisms that contribute to perineural invasion. Highlighting the key events of perineural invasion is important for the identification and testing of novel therapies for oral cancer with perineural invasion.

**Abstract:**

The most common oral cavity cancer is squamous cell carcinoma (SCC), of which perineural invasion (PNI) is a significant prognostic factor associated with decreased survival and an increased rate of locoregional recurrence. In the classical theory of PNI, cancer was believed to invade nerves directly through the path of least resistance in the perineural space; however, more recent evidence suggests that PNI requires reciprocal signaling interactions between tumor cells and nerve components, particularly Schwann cells. Specifically, head and neck SCC can express neurotrophins and neurotrophin receptors that may contribute to cancer migration towards nerves, PNI, and neuritogenesis towards cancer. Through reciprocal signaling, recent studies also suggest that Schwann cells may play an important role in promoting PNI by migrating toward cancer cells, intercalating, and dispersing cancer, and facilitating cancer migration toward nerves. The interactions of neurotrophins with their high affinity receptors is a new area of interest in the development of pharmaceutical therapies for many types of cancer. In this comprehensive review, we discuss diagnosis and treatment of oral cavity SCC, how PNI affects locoregional recurrence and survival, and the impact of adjuvant therapies on tumors with PNI. We also describe the molecular and cellular mechanisms associated with PNI, including the expression of neurotrophins and their receptors, and highlight potential targets for therapeutic intervention for PNI in oral SCC.

## 1. Introduction

Head and neck cancers are the sixth most common cancers in the United States and responsible for nearly 2% of all deaths related to cancer [1]. Approximately 95% of all head and neck cancers are squamous cell carcinoma (SCC); of which, oral cavity SCC is the most common (excluding non-melanoma cutaneous cancers) [2]. The incidence of oral cavity cancer has gradually increased over the past 20 years [3]. Globally, there were more than 375,000 new cases of oral cavity cancers (including cancers of the lip) and more than 175,000 deaths in 2020 [4].

According to the scientific literature, up to 80% of oral cavity SCC demonstrate perineural invasion (PNI) [5,6,7,8,9,10]. PNI occurs when a tumor invades the perineurium space that surrounds a nerve; it is a significant pathological feature in oral cavity SCC because it is associated with pain and multiple cranial neuropathies affecting important functions, such as cutaneous and mucosal sensation, facial mobility, speech, and swallowing. Specifically, PNI has poor prognostic indications in oral cavity SCC and is associated with decreased rate of overall survival, increased rate of local recurrence and regional metastasis, and increased disease-specific mortality [11,12,13,14,15]. Currently, there are no target-directed therapies for PNI in patients with oral cavity SCC, in part because the exact molecular mechanisms of PNI are unknown.

Traditionally, cancer is thought to invade nerves by entering the path of least resistance in the perineural space [16]. However, recent studies suggest that PNI is more complex, involving reciprocal signaling between the tumor itself and the Schwann cells and neurons that compose the nerve [17,18,19,20,21,22]. In this narrative review, we discuss the clinical impact of PNI in oral cavity SCC and the molecular and cellular mechanisms contributing to PNI, including potential roles for Schwann cells. We also provide potential avenues for therapeutic intervention that may alter the progression of PNI in patients with oral cavity SCC.

## 2. Clinical Overview of Oral Squamous Cell Carcinoma

### 2.1. Clinical Presentation

**Figure 1:**
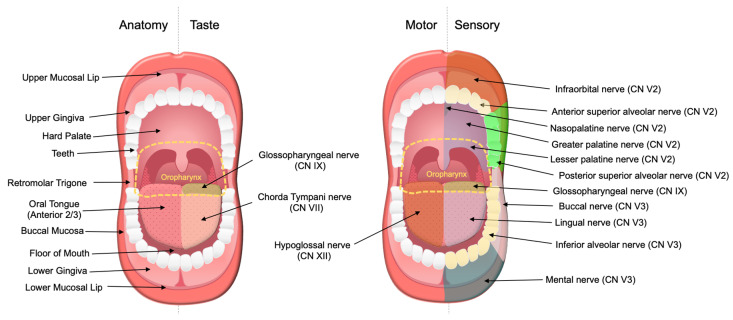
Nerves affected by perineural invasion in oral cavity cancer. The oral cavity cancer site consists of several subsites: oral tongue (anterior 2/3), floor of mouth, hard palate, retromolar trigone, buccal mucosa, upper and lower mucosal lip, and gingiva. The oral tongue (anterior 2/3) receives taste innervation from the chorda tympani (CN VII). The posterior 1/3 of the oral tongue belongs to the oropharynx and receives taste and sensory innervations by the glossopharyngeal nerve (CN IX). Motor innervation of the tongue is primarily supplied by the hypoglossal nerves (CN XII). Sensory innervation of the oral cavity is supplied by the maxillary (CN V2) and mandibular (CN V3) nerves. Perineural invasion in oral cavity cancer may involve any of the aforementioned nerves.

Patients with oral cavity SCC frequently present with white or red mucosal changes referred to as leukoplakia or erythroplakia, respectively. These lesions can involve the oral tongue (anterior 2/3), floor of mouth, hard palate, buccal mucosa, retromolar trigone, and upper and lower mucosal lip, and gingiva [23]. Lesions may also present as a lump, a thickening, or persistent sores or ulcers that fail to resolve [24]. Patients with oral cavity cancer can have severe oral pain, loosening of the teeth, changes in taste, tongue weakness or atrophy, trouble chewing or swallowing, difficulty moving the tongue or jaw, or changes in their ability to speak or articulate words [25]. When oral cavity SCC is associated with PNI, patients may report numbness of the tongue, lip, or other areas of the mouth. Because the oral cavity is richly innervated (Figure 1), PNI in oral cavity SCC can also present as pain, paresthesia, dysesthesia, or motor deficits of cranial nerves V, VII, X, and XII [26,27]. Other symptoms and signs include otalgia, blood in oral secretions, neck mass, and unintentional weight loss.

### 2.2. Diagnosis

The first steps in diagnosing oral cavity SCC are obtaining a complete history and performing a head and neck physical exam. Eliciting risk factors is an important part of the clinical history as tobacco use and alcohol consumption are significant risk factors for the development of oral cavity SCC. In fact, alcohol, smoked tobacco, and smokeless tobacco use have been shown to be synergistic [28]. Outside the United States, another significant risk factor to consider is betel quid (paan), which is frequently chewed in parts of India and southeast Asia. Betel quid is often mixed with other carcinogens such as tobacco and crushed areca nut (also known as betel nut). When combined with smoking tobacco and alcohol, betel quid also has a synergistic effect on the risk of oral cancer occurrence [29].

A head and neck physical exam should encompass: (1) visual inspection and palpation of the oral cavity lesion to define extent of the lesion, (2) cranial nerve exam to assess for signs of PNI, and (3) palpation for cervical lymphadenopathy to assess for potential regional metastasis. A flexible fiberoptic laryngoscopy can help determine the full extent of lesions, rule out other primary lesions in the upper aerodigestive tract, and evaluate airway for operative planning [30].

Although there is a plethora of benign lesions that may present in a similar fashion (including but not limited to oral lichen planus, hairy leukoplakia, aphthous ulcers, pyogenic granulomas, and oral papilloma), persistent lesions that fail to resolve spontaneously or progressively worsen over a period of weeks should be biopsied to rule out malignancy [31,32]. If patients also present with palpable neck masses, needle aspiration with cytologic analysis may be diagnostic.

Computed tomography (CT) with contrast is a commonly ordered imaging modality that evaluates the extent of the oral cavity tumor, presence of lymphadenopathy, and involvement of nearby critical structures such as major cervical vasculature. However, superficial mucosal lesions of the oral cavity may not be evident on radiographic imaging. CT scans are advantageous for detecting osseous involvement, particularly cortical erosions of the mandible and maxilla, with a sensitivity of up to 80% [33,34]. Bone erosion involving cranial nerve canals and widening of cranial nerve foramina suggest the presence of PNI [35].

**Figure 2:**
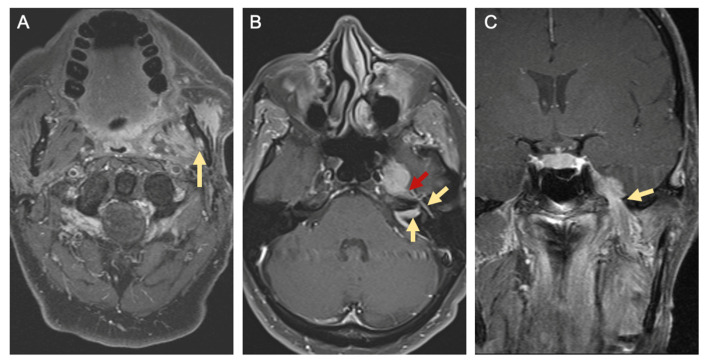
Radiographic signs of perineural spread. T1-weighted magnetic resonance (MR) images with gadolinium from a patient with left buccal squamous cell carcinoma and perineural invasion. (**A**) Axial image showing enlargement and enhancement of the left inferior alveolar canal (yellow arrow). (**B**) Axial image showing enhancement of the intracanalicular and tympanic segments of the facial nerve (yellow arrows) as well as the greater superficial petrosal nerve (red arrow). (**C**) Coronal image showing enlargement and tumor involvement of the mandibular branch of the left trigeminal nerve at the foramen ovale (yellow arrow).

Magnetic resonance (MR) imaging is better at characterizing soft tissue involvement of the head and neck. Hybrid methods of MR imaging may even be useful in detecting lymph node micro-metastasis [34]. Although MR imaging with and without gadolinium is less specific for detecting osseous erosion than CT, foraminal enlargement, replacement of normal fat within the neural foramen, nerve enlargement, and nerve enhancement on MR imaging are key signs of PNI [33,36]. CT of the chest with or without contrast or positron emission tomography with 2-deoxy-2-[fluorine-18]fluoro-D-glucose integrated with CT (^18^F-FDG PET/CT) are tests utilized for detecting distant metastases in patients with advanced nodal disease or identifying primary lung cancers in smokers [35]. Figure 2 demonstrates radiographic evidence of PNI on MR imaging in a patient with left buccal SCC.

At a minimum, diagnosis and staging of oral SCC entails examination with manual palpation, flexible fiberoptic laryngoscopy, biopsy of the lesion, and appropriate imaging [37]. Panendoscopy, involving direct laryngoscopy, bronchoscopy, and esophagoscopy, can help in defining tumor extension into other subsites and identifying synchronous primary tumors, particularly in patients that drink alcohol and smoke tobacco [38,39].

### 2.3. Staging

The American Joint Committee on Cancer (AJCC) Staging of Oral Cavity Cancers (8th edition) is summarized in Table 1 [40]. Greatest tumor dimension, depth of invasion, and extracapsular nodal extension are important features in oral cavity cancer staging, because of their prognostic value in determining disease-specific survival [41,42,43]. Although PNI has been associated with worse disease-specific survival [44,45], it has not been incorporated into the staging of oral cavity SCC.

### 2.4. Treatment Overview

The main treatment options for patients with oral cavity SCC are surgery, radiation therapy, and chemotherapy. Based on the National Comprehensive Cancer Center Guidelines, treatment of oral cavity SCC depends largely on the clinical stage of the cancer and the presence of adverse pathological features [46]. Adverse features include ENE, positive margins, close margins (less than 5 mm), pT3 or pT4 primary tumors, pN2 or pN3 nodal disease, nodal disease in levels IV or V, vascular invasion, lymphatic invasion, and PNI.

Although localized tumors (T1–2, N0) can be treated with either surgical resection or definitive radiotherapy, surgery is generally preferred. Because of the high rate of osteoradionecrosis of the mandible, definitive radiotherapy with external beam radiation is generally reserved for patients who are poor surgical candidates (with significant medical co-morbidities) or when surgical resection would lead to unacceptable severe functional impairment [47,48,49]. Elective neck dissection involving levels I–III or I–IV (ipsilateral or bilateral depending on the location of the primary tumor) or sentinel lymph node biopsy may also be considered in early-stage tumors [46,50]. More advanced tumors (T3, N0; T1–3, N1–3; T4a, any N) are usually treated with definitive surgical resection of the primary lesion with ipsilateral and/or bilateral neck dissections for levels I–IV [37,46,50]. While post-operative radiotherapy may be considered for advanced tumors with no adverse features, advanced oral cavity SCC with ENE and/or positive margins are generally treated with post-operative concurrent chemoradiation. In the absence of ENE and positive margins, post-operative radiation or chemoradiation are generally considered when other risk factors are present, such as pN2–3 or PNI [46].

Treatment of very advanced tumors without distant metastasis (T4b, any N; unresectable nodal disease) can include: (1) concurrent chemoradiation, (2) induction chemotherapy followed by radiotherapy or chemoradiation, (3) definitive radiotherapy with or without chemotherapy, or (4) palliative radiation or chemotherapy [46]. The most common chemotherapy agent utilized is cisplatin, but other systemic therapies may be considered within and outside clinical trials for early and advanced disease (carboplatin, 5-fluorouracil, taxanes, and cetuximab) [51].

### 2.5. Treatment Considerations

In addition to oncologic resection of the primary tumor with 1 cm clinical margins, surgical resection may also include marginal or segmental mandibulectomy if the tumor abuts the periosteum or invades bone, respectively. A partial or total maxillectomy may be necessary for oral cavity cancers that invade the upper gums or hard palate [52]. Furthermore, clearing nerve margins with PNI may require more extensive resection at the skull base. Reconstruction generally follows the reconstructive ladder, beginning with primary closure, secondary intention, or skin grafts for smaller defects. Larger defects may require local tissue transfers and/or free tissue transfer with or without vascularized bone for the restoration of function and cosmesis [53,54,55].

The need for adjuvant therapy for the primary lesion and neck depends largely on the final pathological evaluation of surgical specimens. Adjuvant radiotherapy or chemoradiation is frequently applied postoperatively to treat microscopic disease and decrease risk of locoregional recurrence. Indications for adjuvant therapy can include ENE, positive or close margins, pT3 or pT4 tumors, pN2 or pN3 nodal disease, lymphovascular invasion, and perineural invasion [46,50]. Dosing for post-operative radiation with intensity-modulated radiation therapy (IMRT) or three-dimensional conformal radiation therapy (3D conformal RT) depends on the risk. High risk areas with adverse features such as positive margins receive 60–66 Gy (2 Gy/fraction), while low-to-intermediate risk areas of suspected subclinical spread may receive 44–50 Gy (2.0 Gy/fraction) to 54–63 Gy (1.6–1.8 Gy/fraction) [46]. Although several chemotherapeutic agents have been used for head and neck SCC, high-dose cisplatin is the preferred chemotherapeutic agent for post-operative chemoradiation for oral cavity SCC [56,57,58]. Other variations of cytotoxic anti-cancer agents have also been used including other platinum-based (carboplatin), taxane-based (docetaxel, paclitaxel), and pyrimidine-based treatments (5-fluorouracil) [51].

Over the past two decades, there have been significant developments in targeted therapies for head and neck SCC, in attempts to improve overall survival and limit side effects such as bone marrow suppression. Epidermal growth factor receptors (EGFR) are cell surface receptors of the ErbB family. EGFRs are overexpressed in up to 90% of head and neck SCC, and over expression of EGFR is associated with poorer disease-free and cause-specific survival outcomes [59,60]. Cetuximab is an anti-EGFR immunoglobulin G1 monoclonal antibody approved by the Food and Drug Administration (FDA) for the treatment of head and neck SCC. Cetuximab is indicated as a radiosensitizer for the treatment of locoregionally advanced head and neck SCC and as a monotherapy for patients with recurrent or metastatic cancer who failed platinum-based chemotherapy [61,62]. However, several studies have found cetuximab in combination with radiotherapy to be inferior overall survival to cisplatin and radiotherapy for the treatment of locoregionally advanced head and neck SCC [63,64].

Head and neck SCC also express a relatively high level of programed cell death-ligand 1 (PD-L1), which is a protein that has an important role in suppressing the adaptive immune system. Thus, immunotherapies have become an important part in the treatment of recurrent and metastatic, head and neck SCC after surgery and adjuvant chemoradiation with platinum-based agents. The immune checkpoint inhibitors, pembrolizumab and nivolumab, were recently approved by the FDA for cisplatin-refractory recurrent or metastatic head and neck SCC. Pembrolizumab is also approved as a first-line therapy for PD-L1+ recurrent or metastatic SCC [65,66]. In a multi-institutional clinical trial, pembrolizumab significantly extended overall survival when compared to standard doses of methotrexate, docetaxel, or cetuximab in patients with head and neck SCC [67]. Similarly, nivolumab was shown to prolong survival in patients with platinum-refractory head and neck SCC when compared to standard therapy, with an approximate 20% increase in 1-year survival rates [68]. The consideration of immunotherapy in head and neck SCC is important because research suggests that PNI may alter immune targets in the tumor microenvironment [69].

## 3. Definitions for Perineural Invasion (PNI)

**Figure 3:**
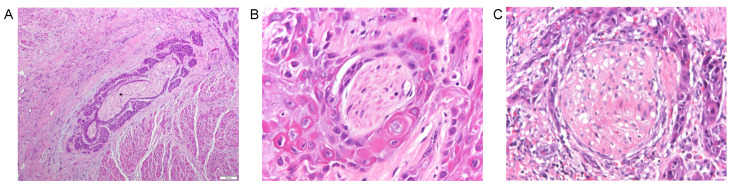
Histopathology of perineural involvement in oral cavity squamous cell carcinoma (SCC). Hematoxylin and eosin-stained histopathological sections of oral cavity SCC demonstrating various types of perineural invasion: (**A**) perineural spread along a longitudinal cut of nerve (10×), (**B**) complete encirclement (60×), and (**C**) incomplete “crescent-like” encirclement of nerves superiorly (40×).

Nerve sheaths are composed of three layers: the endoneurium, perineurium, and epineurium. The endoneurium is the innermost layer that surrounds individual axons and Schwann cells, the perineurium surrounds nerve fascicles, and the epineurium is the outermost layer of dense connective tissue encapsulating nerves [70]. On histopathology, PNI can be broadly defined as the extension of cancer cells around, into, or through a nerve [71]. More specific histopathologic definitions for PNI are tumor involvement within any three layers of the nerve sheath or tumor cells surrounding at least 33% of the nerve’s circumference [72]. However, the determination of PNI on histologic sections remains highly subjective among pathologists, in part from the lack of universal guidelines and the variability on histology of how tumors interact with nerves [73,74]. PNI growth patterns can include complete encirclement, incomplete “crescent-like” encirclement, “onion skin” sandwiching, partial invasion, and neural permeation [75]. In ambiguous cases of nerve involvement, complete or near-total circumferential nerve encirclement, intraneural disease, and perineural tracking seen in adjacent sections is supportive of PNI [76]. Figure 3 illustrates PNI on hematoxylin and eosin staining of histologic sections of oral cavity SCC.

Radiographically, PNI in head and neck cancer can be seen as: (1) direct invasion of nerve, (2) nerve enlargement, (3) nerve enhancement with contrast agents, (4) widening or erosion of skull base foramina, and/or (5) obliteration of the perineural fat at foraminal openings or the pterygopalatine fossa [36]. Some patients with oral cavity cancer will be diagnosed with clinical PNI because they have presenting symptoms and/or radiological features of PNI. Others will have ‘incidental PNI’, when PNI is discovered on histopathological analysis of tumor specimens in the absence of clinical or radiographic features of PNI [77].

## 4. Clinical Impact of Perineural Invasion in Oral Cavity Squamous Cell Carcinoma

### 4.1. Prevalence of Perineural Invasion

Oral cavity SCC has a particular affinity for nerve involvement. In the scientific literature, rates of PNI in oral cavity cancer vary considerably between 6% to 82% [5,6,7,8,9,10]. Although PNI has been associated with worse locoregional recurrence and disease-specific survival [11,12,13,14,15,78], PNI has not been incorporated into the AJCC tumor staging criteria in oral cavity SCC [11,12,13,14,15,78]. In the AJCC Staging Manual, PNI upstages cutaneous SCC of the head and neck to T3 disease, regardless of tumor size or DOI [79]. However, the National Comprehensive Cancer Network lists PNI as an adverse feature in oral cavity cancer that warrants adjuvant treatment with radiotherapy or chemoradiation. In this section, we will discuss the relationship of PNI on locoregional control and survival and review the literature on how adjuvant therapy may affect outcomes in oral cavity SCC with PNI.

### 4.2. Impact of Perineural Invasion on Locoregional Control and Survival

PNI has been repeatedly shown to be a poor prognosticator in oral SCC. In a retrospective case-control study at a single tertiary care center that compared the clinical and histopathological features of 17 oral cavity SCC patients with PNI to controls matched for age, sex, and tumor stage, Laske et al. found that PNI was associated with a 30.3% and 33.3% reduction in 5-year overall and recurrence-free survival, respectively. The 5-year recurrence-free survival rates for early stage (stages I–II) and advanced stage (stages III–IV) oral cavity SCC with PNI were 60.0% and 41.7%, respectively. This is in comparison to controls without PNI that had 5-year recurrence-free survival rates of 94.1% and 73.5% for early and advanced stage oral SCC, respectively [44]. A larger retrospective cohort study investigating the prognostic features of oral and oropharyngeal SCC in 634 patients at a single institution in Mexico found that PNI, when defined as tumor cells invading ≥1 mm of nerve trunk, was significantly associated with locoregional disease recurrence on bivariate analysis. This significance remained on multivariate analysis with a hazard ratio of 8.28. Mean follow-up time was 3.02 years [80]. Similarly, Zanoni et al. retrospectively studied disease features impacting the survival outcomes of 2082 patients with oral cavity SCC who received surgical treatment at a tertiary care center between 1985–2015, with a mean follow-up time of 37.6 months. They found that PNI was present in 24% of patients and was an independent predictor of 5-year overall (risk ratio [RR] 1.26, 95% CI 1.05–1.51; *p =* 0.012) and disease-specific survival (RR 1.36, 95% CI 1.03–1.79; *p =* 0.028) on multivariate analysis [45].

The effect that PNI has on survival is also evident in early tumor (T) stages of disease. Low et al. conducted a retrospective study evaluating 121 patients with AJCC 7th edition T1N0 oral cavity SCC who underwent surgical treatment of disease at a single Australian institution with an average of 3.2 years follow-up. They found that PNI was associated with significantly decreased locoregional control on univariate Kaplan-Meier analysis, with rates of 92% and 53% for absence and presence of PNI, respectively. Although this study was limited in that only five patients had PNI, PNI was almost always observed in those with multiple adverse pathologic features such as lymphovascular invasion and poor tumor differentiation. In this study, the presence of two or more of these features portended significantly increased rates locoregional failures on univariate survival analysis [81]. In a larger study where 442 patients with oral SCC were classified as having pathologic T1 or T2 disease based on AJCC 7th edition staging criteria, a retrospective review of a prospectively maintained database in India revealed that 30 patients with T1 disease and 81 with T2 disease had PNI. On survival analysis, median follow-up time was 25 months and PNI was independently predictive of worse overall survival on multivariable analysis (odds ratio [OR] 2.03, 95% CI 1.06–3.89; *p =* 0.032). When considering AJCC 8th edition tumor staging and PNI, Kaplan-Meier 5-year overall survival rates for T1 disease without and with PNI were 90% and 75%, respectively, while that for T2 disease without and with PNI were 77% and 50%, respectively. These differences in survival with PNI became even more significant when corrected for depth of invasion (*p* < 0.001) [82].

Although the effect of PNI on predicting poor survival is well-established, the effects of PNI are not homogenous and are dependent on various histopathological definitions of PNI. Laske et al. also found that when patients were separated by histopathological subtype of PNI, where type A was defined as intraneural invasion (*n* = 14) and Type B was defined as circumferential growth of tumor around nerve (*n* = 12), the approximate 30% decrease in 5-year recurrence-free survival persisted for both groups when compared to non-PNI controls, but there was no significant difference in survival between subtypes [44]. Meanwhile, a retrospective study including 229 patients with oral SCC and PNI who underwent radical surgery at a Taiwanese hospital were classified as having either intratumoral (*n* = 153) or extratumoral (*n* = 76) PNI, if diseased nerves were ≤0 mm or >0 mm away from the primary tumor site, respectively. Extratumoral PNI disease resulted in worse 5-year locoregional control (63.7% vs. 79.5%), disease-free survival (53.8 vs. 72.9%), and overall survival (54.1% vs. 72%), when compared to intratumoral PNI on Kaplan-Meier univariate analysis. This significance persisted on multivariate analysis, supporting that extratumoral PNI is a more aggressive feature of disease than PNI occurring within the main tumor specimen [83]. Similarly, Caponio et al. conducted a retrospective review of patients with oral tongue SCC to assess relationships between unifocal or multifocal PNI, intratumoral or peritumoral PNI, and survival. They found that intratumoral PNI was associated with lymph node metastasis, while multifocal PNI, regardless of intra- or peritumoral pattern, predicted poor disease specific survival [84].

### 4.3. Surgical Resection and Elective Neck Dissection for Perineural Invasion

PNI may also predict nodal spread of disease, impacting surgical decision making in a clinically or pathologically node-negative neck. Chatzistefanou et al. sought to determine the impact of PNI-positive oral SCC on neck management in a retrospective case control study. At a tertiary care cancer center, 39 patients with PNI-positive disease were compared to patients with PNI-negative tumors and matched for features such as age, T stage, negative surgical margins, lack of lymphovascular invasion, and pN0 disease for a mean follow-up time of 42.7 months. PNI independently predicted lymph node metastasis (OR 3.97; *p* = 0.005) and regional recurrence (OR 5.59, 95% CI 0.57–271.08; *p* = 0.009) on multivariate analysis, and elective neck dissection was associated with lower rates of regional recurrence in PNI positive patients (OR 0.025, 95% CI 0.001–0.645; *p* = 0.026) [85]. These findings indicate that elective neck dissection is beneficial for disease management in patients with pN0 disease irrespective of T stage.

Other studies considered the impact of PNI in predicting cervical nodal disease in early, T1 stage tumors. For example, Nguyen et al. conducted a retrospective cohort study assessing 70 patients with AJCC 8th edition T1N0 oral SCC who underwent either elective neck dissection or neck observation at a single institution. In this cohort, 10 patients had PNI, and all patients were followed for at least 24 months, with a median of 55 months follow-up. They found that the presence of PNI was associated with a higher risk of nodal disease (*p* = 0.002) on categorical analysis, and PNI significantly affected 2-year disease-free survival on multivariate analysis (hazard ratio [HR] 3.15, 95% CI 1.21–8.19; *p* = 0.018), suggesting that patients with PNI-positive disease might benefit from an elective neck dissection [86]. Feng et al. conducted a multicenter, multinational, ambispective cohort study of 283 patients with AJCC 8th edition clinical T1N0 oral SCC and at least 2 years of follow-up to determine what disease features predicted cervical metastasis. Although only eight patients were positive for PNI, its presence resulted in a higher risk of neck metastasis on Cox regression univariate analysis when compared to PNI-negative disease (HR 4.25, 95% CI 1.29–13.96; *p* = 0.017). On multivariate analysis, however, only DOI and tumor grade predicted neck metastasis [87]. Although these studies are promising in guiding the management of T1N0 oral SCC patients with PNI, they are limited by small numbers of PNI-positive patients.

### 4.4. Adjuvant Radiotherapy or Chemoradiation for Perineural Invasion

Despite the evidence showing that PNI is a poor prognostic indicator and may necessitate escalation of surgical treatment in oral SCC, the indication for adjuvant radiotherapy in the presence of isolated PNI is less clear. In a retrospective study from Taiwan, 460 patients with pathologic T1–3N0 (AJCC 5th edition) oral SCC were reviewed. Of these, 68 had PNI, and 24 patients with PNI-positive disease underwent adjuvant radiotherapy. Of those with PNI-positive disease, the authors found no significant differences in 5-year local control or overall survival when comparing those treated with or without adjuvant radiotherapy on Kaplan-Meier analysis [88].

However, a retrospective study of over 1500 patients with oral SCC at a tertiary cancer center, 310 of whom were PNI-positive, found that although patients with PNI and early stage N0 oral SCC had significantly poorer disease-free and overall survival (HR 2.79 and 2.54, respectively) on multivariate analysis, the addition of adjuvant radiotherapy led to significantly improved survival (HR 2.9, *p* = 0.022) [89]. In another study, Rajappa et al. retrospectively analyzed the records of 169 patients with PNI-positive, primary, T1-T2, N0 oral cavity SCC in India. They defined PNI as evidence of a tumor within any layer of the nerve sheath. A total of 118 PNI-positive patients received adjuvant radiotherapy and median follow-up was 45 months. Those treated with postoperative radiotherapy had significantly less nodal recurrence than those treated with observation (8 versus 10 patients, respectively; *p* = 0.013), but there were no differences in rates of local, locoregional, or overall recurrence between groups. Additionally, adjuvant treatment was associated with better disease-free survival in Kaplan–Meier analysis (*p* = 0.047), but there was no significant overall survival benefit (*p* = 0.54) [90]. A 2013 study by Chinn et al. retrospectively investigated 88 patients with pathologic N0 oral SCC treated at a tertiary care center found similarly mixed results. In total, 20 patients had PNI-positive disease and 14 of them were treated with adjuvant radiotherapy. On univariate survival analysis, PNI-positive patients who received adjuvant radiotherapy had a significantly improved disease-free interval of 6.5 years when compared to their non-adjuvant therapy cohort’s survival of 1.7 years (*p* = 0.012). Locoregional control was similarly improved (6.7 versus 1.9 years, respectively; *p* = 0.047). However, there were no differences in overall and disease-specific survival. An analysis of specific histopathologic features of PNI in relation to outcomes was also performed but was inconclusive and underpowered [91].

Adjuvant chemotherapy, in addition to radiotherapy, is another treatment option that is not fully characterized in the context of PNI-positive oral SCC. One retrospective study compared the outcomes of 34 patients with oral SCC who were treated with adjuvant radiotherapy (64.7% with PNI) to 34 who were treated with concurrent chemoradiation (61.8% with PNI). Median follow up was 86.4 months, and results revealed that postoperative concurrent chemoradiation led to significantly improved 5-year overall survival when compared to radiotherapy alone (67.2% versus 35.3%, respectively; *p* = 0.018). Recurrence-free survival for the chemoradiation group was also higher, at 75.4%, when compared to the radiation only group, at 42.6% (*p* < 0.01). This study did not directly compare the effect of PNI on outcomes between groups [92]. In a multi-institutional study that included 1282 patients with oral SCC, 196 were treated with adjuvant chemoradiation, of whom 128 had PNI. On multivariate analysis, disease-free survival was significantly worse in those with PNI (HR 3.08, 95% CI 1.71–5.53, *p* < 0.001) and significantly better in those receiving at least 200 mg/m^2^ adjuvant cisplatin (HR 0.951, 95% CI 0.91–0.99; *p* = 0.007), with median survival approximately double that of the lower dose cohort [93]. To gain further insight into how radiation and chemotherapy may affect oral SCC tumors with PNI, the mechanisms of PNI must be elicited, the histopathological definition of PNI should be standardized, and prospective investigations are warranted.

## 5. Potential Mechanisms of Perineural Invasion

### 5.1. Cellular Observations in Perineural Invasion

PNI has previously been described with a broad definition, encompassing everything from direct tumor invasion into every layer of the peripheral nerve sheath to tumor abutment of cancer. One of the most cited definitions for PNI offered by Batsakis in 1985, defined PNI as tumor cell invasion in, around, and through the nerves [71]. Given this frame of reference, PNI was thought to be a unilateral process dependent upon cancer invasion, with a tumor invading the perineural space through the path of least resistance [72]. However, we are beginning to understand that the perineural microenvironment which involves constituents of nerve may play a significant role in facilitating PNI.

PNI is more likely a complex and dynamic process that relies on reciprocal signaling between the tumor and components of the nerve (e.g., Schwann cells and neurons). Recent evidence suggests that SCC can communicate with nerves long before physical contact between cell types occur [17,18,21,22,27,94,95]. Head and neck SCC may secrete neurotrophic factors into the local environment that are detected by Schwann cells and neurons. Once detected, Schwann cells and neurons can secrete other neurotrophic factors that lead to downstream events, promoting cancer cell invasion and neurite outgrowth. The interactions of secreted neurotrophins with their high affinity receptors between cancer and nerve is a new area of interest in the pathogenesis of PNI [8,21,22,96].

Schwann cells may also play an important role in the development of PNI in cancer [17,18,97]. Schwann cells are the predominant glial cell of the peripheral nervous system and serve different functions in its myelinating and non-myelinating forms. Myelinating Schwann cells function by insulating neuronal axons but can dedifferentiate into its non-myelinating phenotype, express different proteins such as glial fibrillary acidic protein (GFAP) and execute several tasks to repair nerves after injury. Recent studies with pancreatic cancer suggest that Schwann cells can promote PNI by dedifferentiating, migrating towards cancer, dispersing cancer cells, and trafficking cancer cells back to nerves [17,18].

**Figure 4:**
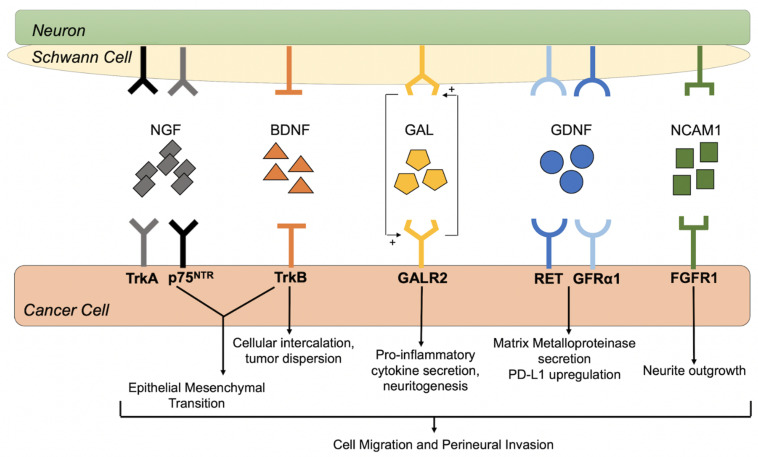
Reciprocal signaling between head and neck SCC and nerve components. Perineural invasion (PNI) is a dynamic and complex process that involves crosstalk between cancer cells, Schwann cells and neurons. This diagram depicts the neurotrophins and their high-affinity receptors that may initiate and promote PNI in head and neck SCC.

Discoveries in pancreatic and prostate cancer have led researchers to focus on cellular interactions influencing cell migration, motility, invasion, and epithelial to mesenchymal transition (EMT) to understand the complex nature of PNI [18,98,99,100,101,102]. In particular, the neurotrophins and their high-affinity receptors have been a major area of interest. In the following sections, we will highlight several neurotrophic factors and receptors that have been implicated in PNI in oral cavity SCC, discuss mechanisms of action, and describe potential avenues for therapeutic intervention. Figure 4 illustrates the neurotrophins and receptors that have been associated with PNI in head and neck SCC.

### 5.2. Nerve Growth Factor and Tropomyosine Receptor Kinase A

Nerve growth factor (NGF) is a member of the neurotrophin family of neurotrophic factors that activates two types of membrane receptors: (1) tropomyosine receptor kinase A (TrkA) tyrosine kinases and (2) p75 neurotrophin receptors (p75NTR), a member of the tumor necrosis factor (TNF) receptor family [103]. By binding to its high-affinity receptors, NGF can modulate cell survival, differentiation, proliferation, migration, and invasion, depending on the receptor and cell type. NGF, TrkA, and p75^NTR^ have been shown to be overexpressed in several human solid malignancies [104], and recent evidence supports their role in the pathogenesis of PNI in oral SCC [105,106,107,108].

In a retrospective study, Kolokythas et al. analyzed NGF and TrkA expression in archived tissue from 42 early stage (T1/T2) oral tongue SCC, half of which demonstrated PNI. They found that PNI-positive tumors had significantly higher immunostaining for NGF (*p* = 0.0001) and TrkA (*p* = 0.039), when compared to PNI-negative tumors. In their study, malignant cells from PNI-positive tumors demonstrated strong cytoplasmic expression of NGF and TrkA, while malignant cells from PNI-negative tumors had weak cytoplasmic expression, suggesting that NGF-TrkA signaling may play an important role in PNI in oral SCC [96].

Other investigations have also demonstrated associations between NGF expression and PNI in oral SCC. Shen et al. performed immunohistochemical staining for NGF on 116 oral tongue SCC specimens and found that high NGF expression levels were significantly associated with PNI (*p* = 0.0009) among other factors, including nodal metastasis (*p* = 0.004) and advanced clinical stage (*p* < 0.0001) [109]. Using the Cancer Genome Atlas database, Lin et al. analyzed NGF and TrkA gene expression in head and neck SCC [110]. They found that tumors expressed high gene expression levels of NGF and TrkA, when compared to normal tissues. Although there was no difference in TrkA gene expression between the PNI-positive and PNI-negative tumor specimens, they showed that NGF gene expression was elevated in PNI-positive tumors. The authors also performed immunohistochemistry for TrkA and NGF in 115 head and neck SCC tumors and 50 adjacent normal tissue samples [110]. Similarly, they demonstrated that tumors had higher protein expression levels of NGF and TrkA than matched adjacent tumors. Although high TrkA protein expression was not correlated with PNI status, high NGF protein expression significantly correlated with PNI (*p* = 0.0024), larger tumor size (*p* = 0.0172), and higher pathological grade (*p* = 0.0227). In a similar retrospective investigation assessing NGF expression in diagnostic biopsies of oral carcinoma, Yu et al. demonstrated that NGF expression was associated with nodal metastasis (*p* = 0.036) and PNI (*p* = 0.0005). In addition, they found that PNI was an independent predictor of nodal metastasis (*p* = 0.004) and disease-free survival (*p* = 0.019) [111], which highlights the importance of understanding how NGF promotes the development of PNI in oral SCC.

Because PNI is a poor prognostic factor in oral SCC, some researchers have ventured to elucidate the molecular mechanisms associated with NGF/TrkA and PNI and identify important targets for therapeutic intervention. In a retrospective analysis of 132 tissue sections of oral SCC, Alkhadar et al. demonstrated that tumors with PNI expressed NGF and TrkA with greater frequency than tumors without PNI. In cancer cells, NGF was present as intense staining of cytoplasm and nuclei, while TrkA was found diffusely in the cytoplasm and along the surface membrane. Although nerve tissue also had moderate to strong expression of NGF and TrkA, the authors showed that the expression of NGF was positively correlated with TrkA expression in tumor samples (Spearman’s correlation coefficient = 0.65) [8]. Their findings suggest that NGF/TrkA may facilitate interactions between oral SCC and neurons that could lead to PNI. To further corroborate their findings, Alkhadar et al. performed migration and invasion assays using various oral and salivary cancer cell lines and demonstrated that NGF triggered phosphoinositide-3 kinase (PI3K)/protein kinase B (Akt) signaling to initiate cancer cell migration and dispersion [112]. Furthermore, they showed that Akt inhibition with MK2206 blocked NGF-induced cancer cell migration and dispersion, which lends more evidence to the link between NGF and PNI in oral SCC and reveals potential targets for therapeutic intervention.

Experiments performed by Grille et al. suggested that PI3K/Akt signaling can enhance motility and invasiveness of SCC cell lines by EMT [113]. EMT is a cellular process by which epithelial cells lose their polarity and intercellular adhesive properties, shift toward a mesenchymal phenotype, and become more motile and invasive [110,113,114]. In a series of in vitro assays using real-time polymerase chain reaction and immunofluorescent assays, Lin et al. demonstrated that NGF induces gene and protein expression changes that reflect EMT. The authors also showed that NGF enhanced cancer cell migration and invasion in a transwell assay. Furthermore, blocking TrkA signaling reversed EMT in both in vitro and in vivo models of head and neck SCC. These findings support the theory that NGF/TrkA expression in the perineural niche acts as a potent chemoattractant for cancers cells during the PNI process by triggering EMT and promoting cancer cell invasion. Although there has been more focus on NGF and TrkA as important contributors to PNI in oral SCC, other neurotrophic factors have also been implicated.

### 5.3. Brain Derived Neurotrophic Factor and Tropomyosine Receptor Kinase B

Brain-derived neurotrophic factor (BDNF) is also part of the neurotrophin family. BDNF binds to its high-affinity receptor tropomyosin receptor kinase B (TrkB) and plays a critical role in neuronal survival, morphogenesis, and plasticity [115]. In various malignances, BDNF/TrkB signaling can promote cell survival, cell proliferation, metastasis, and neovascularization. Overexpression of BDNF has been associated with increased invasiveness in several cancers [116,117,118] and implicated in the pathogenesis of PNI in pancreatic cancer [119,120,121,122]. BDNF and TrkB are overexpressed in head and neck SCC and have been implicated in tumor progression and invasiveness in patients with head and neck SCC [123,124,125]. Recent studies have also demonstrated a potential role for BDNF/TrkB signaling in the development of PNI in oral SCC.

Kupferman et al. performed transcriptional profiling and immunohistochemical analysis of BDNF and TrkB in 71 archival head and neck SCC specimens. They found a positive correlation between BDNF and TrkB messenger RNA (mRNA) expression (*p* < 0.005) and significant upregulation of both proteins in >50% of tumor specimens, when compared to normal mucosa. The authors went further to test the effect of BDNF on cell migration and invasion using several head and neck SCC cell lines, which included two oral SCC cell lines (OSC19 and MDA1986). They found that OSC19 and MDA1986 had high expression levels of TrkB, and BDNF treatment enhanced the migratory and invasive properties of the cancer cells. Through a series of experiments in vitro and in vivo, they also showed that BDNF activates TrkB, which leads to upregulation of Akt signaling, induction of EMT, tumor progression, and increased migration and invasion in head and neck SCC [125]. The authors also demonstrated that TrkB knockdown with short-interfering RNA (siRNA) suppressed BDNF-mediated chemotaxis and invasion by the oral SCC cell line, OSC19. In OSC19 cells, stable TrkB inhibition (through retroviral vectors containing a short hairpin RNA (shRNA) targeting TrkB) blocked BDNF-induced EMT and cell migration, further supporting the role of BDNF/TrkB signaling in tumor progression, migration, and invasion in oral SCC.

In another translational research study, Yilmaz et al. analyzed 20 head and neck SCC tumors and found that TrkB was expressed in 30% of tumors and was absent in normal mucosa [123]. To understand the role of TrkB on tumor progression and cell migration, the authors performed viability and wound scratch assays on several head and neck SCC cell lines, respectively. They demonstrated that TrkB inhibition with AZ64 suppressed cell proliferation and inhibited cancer cell migration in both a time- and dose-dependent manner. Dudas et al. also conducted a series of experiments investigating the role of BDNF/TrkB in oral SCC. In their experiments, they co-cultured oral fibroblasts and lingual SCC cells and showed that fibroblasts transformed into carcinoma-associated fibroblasts (CAFs), which is a significant source of BDNF that could facilitate EMT in lingual SCC [126]. Overall, these studies show a clear role for BDNF/TrkB in EMT, migration, and invasion of oral SCC; however, a definitive link between BDNF/TrkB and PNI in oral SCC has not been fully established.

To understand the potential role of TrkB and Schwann cells in PNI, Ein et al. conducted migration and invasion assays by co-culturing human Schwann cells and human tongue SCC (SCC9) in 2D plates and performed time-lapse imaging. Cancer cells preferentially migrated toward Schwann cells and, upon contact, there was significant intercalation and mixing of both cell types. In co-culture, the addition of BDNF did not affect migration of Schwann or cancer cells but enhanced the intercalation of cell types and increased Schwann cell-associated cancer cell dispersion. In contrast, TrkB inhibition with ANA-12 initiated Schwann cell de-differentiation and activation (by upregulation of GFAP) and increased Schwann cell migration towards cancer. TrkB inhibition also reduced intercalation of Schwann and cancer cells, which led to the development of a well-defined border between cell types, resembling a barrier to cancer cell invasion. Although the association between SC de-differentiation with TrkB inhibition and the prevention of cancer cell dispersion is not entirely clear, these findings demonstrate a phenomenon that may be protective against PNI that need further investigation.

### 5.4. Glial Cell-Derived Neurotrophic Factor and Receptors

One of the most well-studied neurotrophic factors is glial cell-derived neurotrophic factor (GDNF), which binds to its receptor GDNF family receptor-alpha 1 (GFRalpha1) and activates its cognate RET (rearranged during transfection) receptor tyrosine kinase [127]. GDNF has been shown to play a major role in the migration of cancer cells and PNI in several cancers. In human pancreatic adenocarcinoma (MiaPaCa-2) cells, GDNF binds to its receptor GFRalpha1, activates the RET co-receptor, and promotes mitogen-activated protein kinase (MAPK) signaling to induce cancer cell migration towards nerve in vitro and animal models of PNI [128,129]. A similar relationship between GDNF, RET signaling, and PNI was also demonstrated in prostate cancer [130]. Furthermore, pre-clinical studies using pyrazolopyrimidine-1 have shown that RET inhibition can suppress nerve invasion toward the spinal cord and prevent paralysis in mice implanted with pancreatic cancer [129]. Several studies investigating head and neck SCC also highlight a role for GDNF in promoting cancer cell migration and PNI.

In a translational investigation, Chuang et al. analyzed GDNF expression in tissue from human oral SCC and normal mucosa. With immunohistochemistry, they found higher GDNF expression in oral cavity SCC, when compared to normal mucosal tissues. With migration assays utilizing human oral SCC lines (SCC4 and HSC3), they also demonstrated that GDNF increased migratory activity of cancer cells through transwell assays in a dose-dependent manner. Through a series of inhibitor, neutralizing antibody, and knockout studies using short interfering RNA (siRNA), the authors show that GDNF-associated cancer cell migration occurs through GFRa1 receptor activation and regulation of matrix metalloproteinase (MMP)-9 and MMP-13 through a MAPK/activator protein-1 (AP-1) pathway [131].

Lin et al. investigated the relationship between GDNF, PD-L1, and PNI in head and neck SCC. Using RNA sequencing data from a large cohort of head and neck SCC tumors in the Cancer Genome Atlas database, they found that tumors with PNI expressed more GDNF mRNA expression than those without PNI. PD-L1 mRNA expression levels were also higher in GDNF-positive samples overall. The authors also conducted immunohistochemistry for GDNF and PD-L1 in 145 paraffin-embedded head and neck SCC tumors and discovered a positive correlation between GDNF and PD-L1 protein expression (r = 0.38, *p* < 0.001) [132]. Tumors were then stratified by PNI status and GDNF expression was found to be significantly higher in tumors with PNI (*p* = 0.0193). When focusing on specific areas with PNI, the tumor cells around the nerve demonstrated strong PD-L1 staining while the nerves expressed strong GDNF staining. Furthermore, the relative PD-L1 expression was significantly elevated in areas of PNI compared to matched tissues without PNI (*p* < 0.001). Lastly, Kaplan–Meier and Cox regression analyses showed that GDNF and PNI were correlated with decreased overall survival, and GDNF was an independent predictor of overall survival in patients with head and neck SCC [132].

Lin et al. also co-cultured dorsal root ganglia with head and neck SCC (HN4) cells in Matrigel and observed cancer cells migrating toward and along neurites in the control group. Treatment with regorafenib (RET inhibitor) reduced cancer invasion along neurites, suggesting that GDNF may have a critical role in PNI in head and neck SCC [132]. To further elucidate this relationship, head and neck SCC cell lines were cultured in Matrigel with dorsal root ganglia-conditioned media, and PD-L1 mRNA and protein expression was quantified. Cancer significantly upregulated PD-L1 expression through JAK2-STAT1 signaling, and inhibition of RET blocked this effect [132]. Overall, the findings of this study suggest that: (1) nerves may secrete GDNF and promote PNI in head and neck SCC, and (2) GDNF can enhance PD-L1 expression around the perineural nice to promote cancer cell evasion from the immune system.

### 5.5. Galanin and Galanin Receptors

Galanin is a neuropeptide that binds G-protein coupled receptors, Galanin receptors 1 (GALR1), 2 (GALR2), and 3 (GALR3). GAL has many neurotrophic and neuroprotective roles and exerts its actions through Akt and ERK signaling pathways [133]. Galanin is expressed in several malignant tumors [134,135,136,137,138] and has a multitude of downstream effects, including cell proliferation and cell death, depending on the receptor and cell type [134,136,137,138,139]. Several studies have investigated a link between galanin, its receptors, and PNI in head and neck SCC.

In a retrospective investigation analyzing head and neck SCC from 67 patients, Pearlstein et al. found that 40% of patients had a loss of heterozygosity (LOH) of chromosome 18q, and patients with LOH of the 18q loci had a worse 2-year survival compared to those without 18q LOH (30% versus 63%; *p* = 0.008) [140]. Although three minimally loss regions were identified in the 18q region in head and neck SCC (*D18S39* in 18q21.1; *D18S61* in 18q22.2; *D18S70* in 18q23), the tumor suppressor genes associated with these regions have not been fully elucidated [141]. The *GALR1* gene is a candidate gene that is mapped to chromosome 18q23, suggesting that alterations in this receptor may promote tumor progression and poor survival [142].

GALR1, GALR2, and GALR3 are expressed in immortalized oral keratinocytes and oropharyngeal SCC cell lines [143,144]. Through competitive inhibition of galanin and GALR1 neutralizing antibodies in these cell lines, Henson et al. showed that GALR1 has anti-proliferative effects in keratinocytes and that GALR2 and/or GALR3 exerts proliferative effects [143]. Using a human oral carcinoma cell line transformed to express GALR1, Kanazawa et al. demonstrated that galanin can bind GALR1 and suppress cell proliferation by activating extracellular-regulated protein kinase (ERK)-1/2, reducing cyclin D1 expression, and increasing cyclin-dependent kinase inhibitors p27(Kip1) and p57(Kip2) [145]. These findings are consistent with other studies that suggest that GALR1 is a tumor suppressor in head and neck SCC.

Using sodium bisulfate DNA sequencing, Misawa et al. showed that loss of galanin was associated with hypermethylation of the galanin promoter region in several head and neck SCC cell lines. In a parallel retrospective investigation of 100 patients with head and neck SCC, Misawa et al. found that hypermethylation of the galanin promoter in tumors significantly decreased disease-specific survival (*p* < 0.0001). They also showed that methylation of galanin and the gene pair galanin-and-GALR1 were independent predictors of recurrence with an odds ratio of 8.95 (95% CI: 2.29–35.03) and 23.84 (95% CI: 2.74–207.17), respectively [146]. The culmination of these findings suggests that down regulation of galanin and GALR1 through DNA hypermethylation are poor prognostic factors and may represent an avenue for potential therapeutic intervention.

Investigations in head and neck SCC suggest that GALR2 is pro-tumorigenic. When compared to oral keratinocytes, GALR2 expression was found to be upregulated in multiple head and neck SCC cell line. To elucidate the effects of GALR2 expression, Banerjee et al. induced head and neck SCC cell lines to overexpress GALR2. Overexpression of GALR2 stimulated cell proliferation and survival through ERK and Akt activation in vitro and promoted tumor growth in vivo [144]. Although this study suggests that GALR2 overexpression has an oncogenic role in head and neck SCC, GALR2 may have an opposing role in p53 mutant head and neck SCC by inducing apoptosis [147].

GALR2 overexpression may also play an important role in regulating PNI in head and neck SCC. Scanion et al. analyzed tumors from athymic mice implanted with oral SCC (OSCC3) cells stably overexpressing GALR2 or the control vector. They found that rats implanted with GALR2-overexpressing tumors developed PNI, while those with the control vector did not. GALR2 overexpression was also associated with more neuronal involvement and poor survival in vivo. Through a series of co-cultures and conditioned media assays, Scanion et al. showed that nerve-derived galanin can activate GALR2 in cancer cells and initiate crosstalk between nerve and cancer. GALR2 activation subsequently induces tumor cells to secrete pro-inflammatory mediators and neuropeptides that promote invasion and PNI through NFATC2 (nuclear factor of activated T cells 2)-mediated cyclooxygenase-2 expression. In a complete feedback loop, galanin secretion from tumor-promoted neuritogenesis. Furthermore, they show that downregulation or blockage of GALR2 or galanin disrupted this nerve-tumor crosstalk to prevent PNI and neuritogenesis [94]. Overall, these studies provide an exciting basis for further investigations of galanin and GALR2 at therapeutic targets for PNI in oral SCC.

### 5.6. Neural Cell Adhesion Molecule 1 and Fibroblast Growth Factor Receptor 1

Neural cell adhesion molecule-1 (NCAM1) is an important glycoprotein that binds to fibroblast growth factor receptor-1 (FGFR1) in neurons to induce neurite outgrowth and neuronal migration [148,149]. NCAM overexpression has been associated with PNI in multiple cancers, including prostate cancer, pancreatic cancer, and cutaneous SCC [150,151,152]. In prostate cancer, NCAM is thought to facilitate cancer cell migration towards nerves and perineural invasion in part through nuclear factor kappa B (NFkB) activation [113].

Schwann cells are the principal neuronal support cells, responsible for maintaining nerve homeostasis, facilitating nerve repair following injury, and providing neuronal insulation for signal transduction under normal physiological conditions [153]. Deborde et al. introduced Schwann cells as an important player in the initiation of PNI in pancreatic cancer. In a series of in vitro live imaging assays with pancreatic adenocarcinoma, Schwann cells, and dorsal root ganglia, they demonstrated that Schwann cells can migrate towards cancer, direct cancer cells to migrate toward nerves, and promote invasion in a manner dependent on Schwann cell expression of NCAM1. Schwann cells also intercalated between cancer cells, causing cancer cell dispersion. Furthermore, NCAM1-deficient mice were found to have less PNI than their wild-type counterparts [151]. These findings were critical in showing how the NCAM1/FGFR1 pathway in the tumor microenvironment can activate Schwann cells to facilitate PNI in pancreatic cancer. However, the role of NCAM1 in promoting PNI in head and neck SCC is less clear.

In a retrospective study, Vural et al. performed immunohistochemistry for NCAM on 66 head and neck SCC tumors, of which 41 demonstrated perineural spread. NCAM expression was identified in 93% of tumors with perineural spread, but only 36% of tumors without perineural spread. The difference in NCAM expression between the two groups was significant (*p* < 0.01) [154]. Similarly, McLaughlin analyzed 76 archived head and neck SCC specimens for PNI and NCAM expression. Of the 76 tumors, 37% demonstrated PNI while 50% demonstrated NCAM expression. They also found that NCAM expression was significantly associated with PNI (*p* = 0.002) [155]. In contrast, NCAM expression did not predict neurotropism in a retrospective analysis of cutaneous SCC tumors of the head and neck; however, the study was small (*n* = 14), tumors were of cutaneous origin, and tumor specimens were compared to normal nerves instead of matched controls without PNI [156]. Additional studies are needed to delineate the role of NCAM1 and FGFR1 interaction in oral SCC PNI.

### 5.7. Alternative Pathways Influencing PNI and Tumor Progression

In addition to neurotrophic signaling, there are complementary signaling pathways influencing PNI in other cancers that may have implications in oral cavity SCC. For instance, peripheral nerves secrete serine to induce the growth of pancreatic ductal adenocarcinoma cells. Meanwhile, serine deprivation led to selective secretion of NGF, suggesting that nerve–tumor crosstalk via amino acids may influence PNI [157]. Furthermore, activation of the neurotransmitter N-methyl-d-aspartate receptor by pseudo-tripartite synapses between cancer and nerves has been associated with breast cancer metastasis to the brain [158]. Along those same lines, the interaction between migratory nerve progenitors and prostate tumors in a mouse model has been shown to stimulate nerve infiltration into the tumor and adrenergic neurogenesis [159]. The interactions of secreted factors, receptors, and nerve and Schwann cell states are likely influential in oral cavity SCC with PNI and warrant further investigation.

## 6. Potential Therapeutic Interventions for Perineural Invasion

By elucidating the molecular and cellular mechanisms responsible for PNI in oral SCC, we can investigate how existing cancer therapies (i.e., chemotherapies and radiation) and novel target-directed therapies can block paracrine and Schwann cell-mediated pathways involved in PNI. For example, exposure of dorsal root ganglia to 4 Gy of single fraction radiation significantly reduced GDNF expression and inhibited PNI in co-cultures with pancreatic cancer cells. Similarly, delivering 8 Gy of radiation to the sciatic nerve of mice reduced GDNF expression, decreased PNI, and preserved nerve function in mice implanted with pancreatic cancer cells [160]. Thus, elucidating how radiation therapy can potentially alter the nerve microenvironment in head and neck SCC to reduce the incidence of PNI has many therapeutic implications. Inhibitors that target neurotrophins and their receptors is another avenue of novel therapeutics that may be effective at treating tumors with PNI and prolonging survival in patients with oral SCC [161]. Immunotherapies have made major advances in recent years and may revolutionize treatment of head and neck SCC tumors with PNI. By using targeted therapies to alter the interaction between tumor cells, nerves, and Schwann cells, we may be able to reduce morbidity and improve prognosis in patients with oral cavity SCC.

## 7. Conclusions

PNI is a poor prognostic factor that increases locoregional recurrence and reduces disease-specific and recurrence free-survival in patients with oral cavity SCC. PNI is a dynamic process that entails reciprocal signaling between tumor and nerve constituents, such as the Schwann cells and nerves. Overexpression of several neurotrophins and their receptors have been linked to the development of PNI in head and neck SCC. There is also some evidence that PD-L1 may inhibit immune response in the perineural niche to facilitate PNI in oral SCC. Understanding the molecular and cellular mechanisms of PNI and how adjuvant radiation, chemotherapies, immunotherapies, and novel antagonists of neurotrophins and their receptors can modulate PNI are critical for identifying effective treatments for oral cavity SCC.

## Figures and Tables

**Table 1:** American Joint Committee on Cancer (AJCC) 8th edition staging criteria for oral cavity cancer.

**Primary Tumor (T)**			
TX	Primary tumor cannot be assessed		
Tis	Carcinoma in situ		
T1	Tumor ≤ 2 cm and DOI ≤ 5 mm		
T2	Tumor ≤ 2 cm, DOI > 5 mm and ≤10 mm; or Tumor > 2 cm but ≤4 cm and DOI ≤ 10 mm		
T3	Tumor > 4 cm; or any tumor with DOI > 10 mm but ≤20 mm		
T4a	Moderately Advanced Local Disease: Tumor invades adjacent structures only (e.g., through cortical bone of the mandible or maxilla, or involves the maxillary sinus or skin of the face); or Extensive tumor with bilateral tongue involvement; and/or DOI > 20 mm		
T4b	Very advanced local disease: Tumor invades masticator space, pterygoid plates, or skull base and/or encases the internal carotid artery		
**Clinical Assessment of Regional Lymph Nodes (cN)**			
NX	Regional lymph nodes cannot be assessed		
N0	No regional lymph node metastasis		
N1	Metastasis in a single ipsilateral lymph node, ≤3 cm and ENE−		
N2a	Metastasis in a single ipsilateral node, >3 cm and ≤6 cm and ENE−		
N2b	Metastases in multiple ipsilateral nodes, ≤6 cm and ENE–		
N2c	Metastases in bilateral or contralateral lymph nodes, ≤6 cm and ENE−		
N3a	Metastasis in a lymph node > 6 cm and ENE−		
N3b	Metastasis in any lymph node(s) with ENE+ clinically; or Metastasis in a single ipsilateral node, >3 cm and ENE+; or Metastasis to multiple ipsilateral, contralateral, or bilateral nodes and ENE+		
**Pathologic Assessment of Regional Lymph Nodes (pN)**			
NX	Regional lymph nodes cannot be assessed		
N0	No regional lymph node metastasis		
N1	Metastasis in a single ipsilateral lymph node, ≤3 cm and ENE−		
N2a	Metastasis in a single ipsilateral node, ≤3 cm and ENE+; or Metastasis in a single ipsilateral node > 3 cm and ≤6 cm and ENE−		
N2b	Metastases in multiple ipsilateral nodes, ≤6 cm and ENE–		
N2c	Metastases in bilateral or contralateral lymph nodes, ≤6 cm and ENE−		
N3a	Metastasis in a lymph node > 6 cm and ENE−		
N3b	Metastasis in a single ipsilateral node > 3 cm with ENE+; or Metastasis to multiple ipsilateral, contralateral, or bilateral nodes, any with ENE+; or Metastasis to a single contralateral node of any size and ENE+		
**Distant Metastasis (M)**			
M0	No distant metastases		
M1	Distant metastases		
**AJCC Prognostic Stage Groups**			
Stage 0	Tis	N0	M0
Stage I	T1	N0	M0
Stage II	T2	N0	M0
Stage III	T3	N0	M0
	T1–T3	N1	M0
Stage IVa	T4a	N0	M0
	T4a	N1	M0
	T1–T4a	N2	M0
Stage IVb	Any T	N3	M0
	T4b	Any N	M0
Stage IVc	Any T	Any N	M1

Abbreviations: DOI, depth of invasion; ENE, extranodal extension.

## Abbreviations

AJCC	American Joint Committee on Cancer
AP-1	Activator protein-1
BDNF	Brain-derived neurotrophic factor
CI	Confidence interval
cN	Clinical nodal
CT	Computed tomography
DNA	Deoxyribonucleic acid
DOI	Depth of invasion
EGFR	Epidermal growth factor receptors
EMT	Epithelial to mesenchymal transition
ENE	Extracapsular nodal extension
ERK	Extracellular-regulated protein kinase
FDA	Food and Drug Administration
FGFR	Fibroblast growth factor receptor
GALR	Galanin receptor
GDNF	Glial cell-derived neurotrophic factor
GFAP	Glial fibrillary acidic protein
GFRalpha1	GDNF family receptor-alpha 1
HPV	Human papilloma virus
HR	Hazard ratio
IMRT	Intensity-modulated radiation therapy
LOH	Loss of heterozygosity
M	Metastasis
MAPK	Mitogen-activated protein kinase
MMP	Matrix metalloproteinase
MR	Magnetic resonance
mRNA	Messenger ribonucleic acid
NCAM	Neural cell adhesion molecule
NFATC	Nuclear factor of activated T cells
NGF	Nerve growth factor
OR	Odds ratio
p75NTR	p75 neurotrophin receptors
PD-L1	Programed cell death -ligand 1
pN	Pathological nodal
PNI	Perineural invasion
RET	REarranged during Transfection
SCC	Squamous cell carcinoma
shRNA	Short hairpin ribonucleic acid
T	Tumor
TNF	Tumor necrosis factor
TrkA	Tropomyosin receptor kinase A
TrkB	Tropomyosin receptor kinase B

## References

[1] National Cancer Institute (US) SEER Cancer Stat Facts: Oral Cavity and Pharynx Cancer. https://seer.cancer.gov/statfacts/html/oralcav.html.

[2] Chi A.C., Day T.A., Neville B.W. (2015). Oral cavity and oropharyngeal squamous cell carcinoma-an update. CA Cancer J. Clin..

[3] American Cancer Society (2021). Cancer Facts & Figures 2021.

[4] Sung H., Ferlay J., Siegel R.L., Laversanne M., Soerjomataram I., Jemal A., Bray F. (2021). Global Cancer Statistics 2020: GLOBOCAN Estimates of Incidence and Mortality Worldwide for 36 Cancers in 185 Countries. CA Cancer J. Clin..

[5] Carter R.L., Foster C.S., A Dinsdale E., Pittam M.R. (1983). Perineural spread by squamous carcinomas of the head and neck: A morphological study using antiaxonal and antimyelin monoclonal antibodies. J. Clin. Pathol..

[6] Soo K.-C., Carter R.L., O’Brien C.J., Barr L., Bliss J.M., Shaw H.J. (1986). Prognostic implications of perineural spread in squamous carcinomas of the head and neck. Laryngoscope.

[7] Kurtz K.A., Hoffman H.T., Zimmerman M.B., Robinson R.A. (2005). Perineural and Vascular Invasion in Oral Cavity Squamous Carcinoma: Increased Incidence on Re-review of Slides and by Using Immunohistochemical Enhancement. Arch. Pathol. Lab. Med..

[8] Alkhadar H., Macluskey M., White S., Ellis I. (2020). Perineural invasion in oral squamous cell carcinoma: Incidence, prognostic impact and molecular insight. J. Oral Pathol. Med..

[9] Wei P.-Y., Li W.-Y., Tai S.-K. (2019). Discrete Perineural Invasion Focus Number in Quantification for T1-T2 Oral Squamous Cell Carcinoma. Otolaryngol. Head Neck Surg..

[10] Schmitd L., Scanlon C., D’Silva N. (2018). Perineural Invasion in Head and Neck Cancer. J. Dent. Res..

[11] Brown B., Barnes L., Mazariegos J., Taylor F., Johnson J., Bs R.L.W. (1989). Prognostic factors in mobile tongue and floor of mouth carcinoma. Cancer.

[12] Aivazian K., Ebrahimi A., Low T.-H.H., Gao K., Clifford A., Shannon K., Clark J.R., Gupta R. (2015). Perineural invasion in oral squamous cell carcinoma: Quantitative subcategorisation of perineural invasion and prognostication. J. Surg. Oncol..

[13] Tai S.-K., Li W.-Y., Yang M.-H., Chang S.-Y., Chu P.-Y., Tsai T.-L., Wang Y.-F., Chang P.M.-H. (2012). Treatment for T1-2 Oral Squamous Cell Carcinoma with or Without Perineural Invasion: Neck Dissection and Postoperative Adjuvant Therapy. Ann. Surg. Oncol..

[14] Fagan J.J., Collins B., Barnes L., D’Amico F., Myers E.N., Johnson J.T. (1998). Perineural Invasion in Squamous Cell Carcinoma of the Head and Neck. Arch. Otolaryngol. Head Neck Surg..

[15] Kim R.Y., Helman J.I., Braun T.M., Ward B.B. (2019). Increased Presence of Perineural Invasion in the Tongue and Floor of the Mouth: Could It Represent a More Aggressive Oral Squamous Cell Carcinoma, or Do Larger Aggressive Tumors Cause Perineural Invasion?. J. Oral Maxillofac. Surg..

[16] Binmadi N.O., Basile J.R. (2011). Perineural invasion in oral squamous cell carcinoma: A discussion of significance and review of the literature. Oral Oncol..

[17] Azam S.H., Pecot C.V. (2016). Cancer’s got nerve: Schwann cells drive perineural invasion. J. Clin. Investig..

[18] Deborde S., Wong R.J. (2017). How Schwann cells facilitate cancer progression in nerves. Cell. Mol. Life Sci..

[19] Roger E., Martel S., Bertrand-Chapel A., Depollier A., Chuvin N., Pommier R., Yacoub K., Caligaris C., Cardot V., Chauvet V. (2019). Schwann cells support oncogenic potential of pancreatic cancer cells through TGFβ signaling. Cell Death Dis..

[20] Huang T., Fan Q., Wang Y., Cui Y., Wang Z., Yang L., Sun X., Wang Y. (2020). Schwann Cell-Derived CCL2 Promotes the Perineural Invasion of Cervical Cancer. Front. Oncol..

[21] Ein L., Bracho O., Mei C., Patel J., Boyle T., Monje P., Fernandez-Valle C., Bas E., Thomas G., Weed D. (2019). Inhibition of tropomyosine receptor kinase B on the migration of human Schwann cell and dispersion of oral tongue squamous cell carcinoma in vitro. Head Neck.

[22] Ein L., Mei C., Bracho O., Bas E., Monje P., Weed D., Sargi Z., Thomas G., Dinh C. (2019). Modulation of BDNF–TRKB Interactions on Schwann Cell-induced Oral Squamous Cell Carcinoma Dispersion In Vitro. Anticancer. Res..

[23] The American Cancer Society What Are Oral Cavity and Oropharyngeal Cancers?. https://www.cancer.org/cancer/oral-cavity-and-oropharyngeal-cancer/about/what-is-oral-cavity-cancer.html#references.

[24] PDQ Adult Treatment Editorial Board (2002). Lip and Oral Cavity Cancer Treatment (Adult) (PDQ(R)): Health Professional Version. PDQ Cancer Information Summaries.

[25] The American Cancer Society Medical and Editorial Content Team Signs and Symptoms of Oral Cavity and Oropharyngeal Cancer. https://www.cancer.org/cancer/oral-cavp.

[26] Massey B.T. (2006). Physiology of oral cavity, pharynx, and upper esophageal sphincter. GI Motil. Online.

[27] Bakst R.L., Glastonbury C.M., Parvathaneni U., Katabi N., Hu K.S., Yom S. (2019). Perineural Invasion and Perineural Tumor Spread in Head and Neck Cancer. Int. J. Radiat. Oncol..

[28] Mello F.W., Melo G., Pasetto J.J., Silva C.A.B., Warnakulasuriya S., Rivero E. (2019). The synergistic effect of tobacco and alcohol consumption on oral squamous cell carcinoma: A systematic review and meta-analysis. Clin. Oral Investig..

[29] Petti S., Masood M., Scully C. (2013). The Magnitude of Tobacco Smoking-Betel Quid Chewing-Alcohol Drinking Interaction Effect on Oral Cancer in South-East Asia. A Meta-Analysis of Observational Studies. PLoS ONE.

[30] The American Cancer Society Medical and Editorial Content Team Tests for Oral Cavity and Oropharyngeal Cancers. https://www.cancer.org/cancer/oral-cavity-and-oropharyngeal-cancer/detection-diagnosis-staging/how-diagnosed.html.

[31] Maymone M.B., Greer R.O., Burdine L.K., Dao-Cheng A., Venkatesh S., Sahitya P.C., Maymone A.C., Kesecker J., Vashi N.A. (2019). Benign oral mucosal lesions: Clinical and pathological findings. J. Am. Acad. Dermatol..

[32] Gonsalves W.C., Chi A.C., Neville B.W. (2007). Common Oral Lesions: Part I. Superficial Mucosal Lesions. Am. Fam. Phys..

[33] Trotta B.M., Pease C.S., Rasamny J.J., Raghavan P., Mukherjee S. (2011). Oral Cavity and Oropharyngeal Squamous Cell Cancer: Key Imaging Findings for Staging and Treatment Planning. Radiographics.

[34] Pałasz P., Adamski Ł., Górska-Chrząstek M., Starzyńska A., Studniarek M. (2017). Contemporary Diagnostic Imaging of Oral Squamous Cell Carcinoma–A Review of Literature. Pol. J. Radiol..

[35] Lee H., Lazor J.W., Assadsangabi R., Shah J. (2019). An Imager’s Guide to Perineural Tumor Spread in Head and Neck Cancers: Radiologic Footprints on 18F-FDG PET, with CT and MRI Correlates. J. Nucl. Med..

[36] Chong V.-H. (2010). Imaging of perineural spread in head and neck tumours. Cancer Imaging.

[37] Machiels J.-P., Leemans C.R., Golusinski W., Grau C., Licitra L., Gregoire V. (2020). Squamous cell carcinoma of the oral cavity, larynx, oropharynx and hypopharynx: EHNS–ESMO–ESTRO Clinical Practice Guidelines for diagnosis, treatment and follow-up. Ann. Oncol..

[38] Koo K., Harris R., Wiesenfeld D., Iseli T.A. (2015). A role for panendoscopy? Second primary tumour in early stage squamous cell carcinoma of the oral tongue. J. Laryngol. Otol..

[39] Rodriguez-Bruno K., Ali M.J., Wang S.J. (2011). Role of panendoscopy to identify synchronous second primary malignancies in patients with oral cavity and oropharyngeal squamous cell carcinoma. Head Neck.

[40] Amin M.B., Edge S.B., Greene F., Byrd D.R., Brookland R.K., Washington M.K., Gershenwald J.E., Compton C.C., Hess K.R., Sullivan D.C. (2017). AJCC Cancer Staging Manual.

[41] Ebrahimi A., Gil Z., Amit M., Yen T.-C., Liao C.-T., Chaturvedi P., Agarwal J.P., Kowalski L.P., Kreppel M., Cernea C.R. (2014). Primary Tumor Staging for Oral Cancer and a Proposed Modification Incorporating Depth of Invasion: An international multicenter retrospective study. JAMA Otolaryngol. Neck Surg..

[42] Zanoni D.K., Patel S.G., Shah J.P. (2019). Changes in the 8th Edition of the American Joint Committee on Cancer (AJCC) Staging of Head and Neck Cancer: Rationale and Implications. Curr. Oncol. Rep..

[43] Wreesmann V.B., Katabi N., Ba F.L.P., Montero P.H., Ma J.C.M., Gönen M., Carlson D.L., Ganly I., Shah J.P., A Ghossein R. (2016). Influence of extracapsular nodal spread extent on prognosis of oral squamous cell carcinoma. Head Neck.

[44] Laske R.D., Scholz I., Ikenberg K., Meerwein C., Vital D.G., Studer G., Rössle M., Huber G.F. (2016). Perineural Invasion in Squamous Cell Carcinoma of the Oral Cavity: Histology, Tumor Stage, and Outcome. Laryngoscope Investig. Otolaryngol..

[45] Zanoni D.K., Montero P.H., Migliacci J.C., Shah J.P., Wong R.J., Ganly I., Patel S.G. (2019). Survival outcomes after treatment of cancer of the oral cavity (1985–2015). Oral Oncol..

[46] National Comprehensive Cancer Network Head and Neck Cancers (Version 3.2021). https://www.nccn.org/professionals/physician_gls/pdf/head-and-neck.pdf.

[47] Chinn S., Myers J.N. (2015). Oral Cavity Carcinoma: Current Management, Controversies, and Future Directions. J. Clin. Oncol..

[48] Kuhnt T., Stang A., Wienke A., Vordermark D., Schweyen R., Hey J. (2016). Potential risk factors for jaw osteoradionecrosis after radiotherapy for head and neck cancer. Radiat. Oncol..

[49] Fujiwara R.J., Burtness B., Husain Z.A., Judson B.L., Bhatia A., Sasaki C.T., Yarbrough W.G., Mehra S. (2017). Treatment guidelines and patterns of care in oral cavity squamous cell carcinoma: Primary surgical resection vs. nonsurgical treatment. Oral Oncol..

[50] Koyfman S.A., Ismaila N., Crook D., D’Cruz A., Rodriguez C.P., Sher D.J., Silbermins D., Sturgis E.M., Tsue T.T., Weiss J. (2019). Management of the Neck in Squamous Cell Carcinoma of the Oral Cavity and Oropharynx: ASCO Clinical Practice Guideline. J. Clin. Oncol..

[51] Szturz P., Vermorken J.B. (2020). Management of recurrent and metastatic oral cavity cancer: Raising the bar a step higher. Oral Oncol..

[52] Shanti R.M., O’Malley B.W. (2018). Surgical Management of Oral Cancer. Dent. Clin. N. Am..

[53] Rigby M.H., Taylor S.M. (2013). Soft tissue reconstruction of the oral cavity: A review of current options. Curr. Opin. Otolaryngol. Head Neck Surg..

[54] Patel U.A., Hartig G.K., Hanasono M.M., Lin D.T., Richmon J.D. (2017). Locoregional Flaps for Oral Cavity Reconstruction: A Review of Modern Options. Otolaryngol. Neck Surg..

[55] Haughey B.H., Fredrickson J.M., Lerrick A.J., Sclaroff A., Gay W.D. (1994). Fibular and Iliac Crest Osteomuscular Free Flap Reconstruction of the Oral Cavity. Laryngoscope.

[56] Bachaud J.-M., Cohen-Jonathan E., Alzieu C., David J.-M., Serrano E., Daly-Schveitzer N. (1996). Combined postoperative radiotherapy and Weekly Cisplatin infusion for locally advanced head and neck carcinoma: Final report of a randomized trial. Int. J. Radiat. Oncol..

[57] Bernier J., Cooper J.S., Pajak T.F., Van Glabbeke M., Bourhis J., Forastiere A., Ozsahin E.M., Jacobs J.R., Jassem J., Ang K.-K. (2005). Defining risk levels in locally advanced head and neck cancers: A comparative analysis of concurrent postoperative radiation plus chemotherapy trials of the EORTC (#22931) and RTOG (# 9501). Head Neck.

[58] Cooper J.S., Pajak T.F., Forastiere A.A., Jacobs J., Campbell B., Saxman S.B., Kish J.A., Kim H.E., Cmelak A.J., Rotman M. (2004). Postoperative Concurrent Radiotherapy and Chemotherapy for High-Risk Squamous-Cell Carcinoma of the Head and Neck. N. Engl. J. Med..

[59] Kalyankrishna S., Grandis J.R. (2006). Epidermal Growth Factor Receptor Biology in Head and Neck Cancer. J. Clin. Oncol..

[60] Grandis J.R., Melhem M.F., Gooding W.E., Day R.S., Holst V.A., Wagener M.M., Drenning S.D., Tweardy D.J. (1998). Levels of TGF-α and EGFR Protein in Head and Neck Squamous Cell Carcinoma and Patient Survival. J. Natl. Cancer Inst..

[61] Bonner J.A., Harari P.M., Giralt J., Azarnia N., Shin D.M., Cohen R.B., Jones C.U., Sur R., Raben D., Jassem J. (2006). Radiotherapy plus Cetuximab for Squamous-Cell Carcinoma of the Head and Neck. N. Engl. J. Med..

[62] Vermorken J.B., Trigo J., Hitt R., Koralewski P., Diaz-Rubio E., Rolland F., Knecht R., Amellal N., Schueler A., Baselga J. (2007). Open-Label, Uncontrolled, Multicenter Phase II Study to Evaluate the Efficacy and Toxicity of Cetuximab As a Single Agent in Patients with Recurrent and/or Metastatic Squamous Cell Carcinoma of the Head and Neck Who Failed to Respond to Platinum-Based Therapy. J. Clin. Oncol..

[63] Bauml J.M., Vinnakota R., Park Y.A., Bates S.E., Fojo T., Aggarwal C., Di Stefano J., Knepley C., Limaye S., Mamtani R. (2019). Cisplatin versus cetuximab with definitive concurrent radiotherapy for head and neck squamous cell carcinoma: An analysis of Veterans Health Affairs data. Cancer.

[64] Stokes W.A., Sumner W.A., Breggren K.L., Rathbun J.T., Raben D., McDermott J.D., Gan G., Karam S.D. (2017). A comparison of concurrent cisplatin versus cetuximab with radiotherapy in locally-advanced head and neck cancer: A bi-institutional analysis. Rep. Pract. Oncol. Radiother..

[65] Burtness B., Harrington K., Greil R., Soulières D., Tahara M., de Castro G., Psyrri A., Basté N., Neupane P., Bratland A. (2019). Pembrolizumab alone or with chemotherapy versus cetuximab with chemotherapy for recurrent or metastatic squamous cell carcinoma of the head and neck (KEYNOTE-048): A randomised, open-label, phase 3 study. Lancet.

[66] Kitamura N., Sento S., Yoshizawa Y., Sasabe E., Kudo Y., Yamamoto T. (2020). Current Trends and Future Prospects of Molecular Targeted Therapy in Head and Neck Squamous Cell Carcinoma. Int. J. Mol. Sci..

[67] Cohen E.E.W., Soulières D., Le Tourneau C., Dinis J., Licitra L., Ahn M.-J., Soria A., Machiels J.-P., Mach N., Mehra R. (2019). Pembrolizumab versus methotrexate, docetaxel, or cetuximab for recurrent or metastatic head-and-neck squamous cell carcinoma (KEYNOTE-040): A randomised, open-label, phase 3 study. Lancet.

[68] Ferris R.L., Blumenschein G., Fayette J., Guigay J., Colevas A.D., Licitra L., Harrington K., Kasper S., Vokes E.E., Even C. (2016). Nivolumab for Recurrent Squamous-Cell Carcinoma of the Head and Neck. N. Engl. J. Med..

[69] Yang M.-W., Tao L.-Y., Jiang Y.-S., Yang J.-Y., Huo Y.-M., Liu D.-J., Li J., Fu X.-L., He R., Lin C. (2020). Perineural invasion reprograms the immune microenvironment through cholinergic signaling in pancreatic ductal adenocarcinoma. Cancer Res..

[70] Peltonen S., Alanne M., Peltonen J. (2013). Barriers of the peripheral nerve. Tissue Barriers.

[71] Batsakis J.G. (1985). Nerves and neurotropic carcinomas. Ann. Otol. Rhinol. Laryngol..

[72] Liebig C., Ayala G., Wilks J.A., Berger D.H., Albo D. (2009). Perineural invasion in cancer: A review of the literature. Cancer.

[73] Chi A.C., Katabi N., Chen H.-S., Cheng Y.-S.L. (2016). Interobserver Variation among Pathologists in Evaluating Perineural Invasion for Oral Squamous Cell Carcinoma. Head Neck Pathol..

[74] Yan F., Cheng Y.-S.L., Katabi N., Nguyen S.A., Chen H.-S., Morgan P., Zhang K., Chi A.C. (2021). Interobserver Variation in Evaluating Perineural Invasion for Oral Squamous Cell Carcinoma: Phase 2 Survey Study. Head Neck Pathol..

[75] Varsha B.K., Radhika M.B., Makarla S., Kuriakose M.A., Kiran G.S., Padmalatha G.V. (2015). Perineural invasion in oral squamous cell carcinoma: Case series and review of literature. J. Oral Maxillofac. Pathol..

[76] Dunn M., Morgan M.B., Beer T. (2009). Perineural Invasion: Identification, Significance, and a Standardized Definition. Dermatol. Surg..

[77] Keerthi R., Dutta A., Agarwal S., Kani V., Khatua A. (2018). Perineural Invasion of Oral Squamous Cell Carcinoma: A New Hurdle for Surgeons. J. Maxillofac. Oral Surg..

[78] Conte C.C., Ergin M., Ricci A., Deckers P.J. (1989). Clinical and pathologic prognostic variables in oropharyngeal squamous cell carcinoma. Am. J. Surg..

[79] Lydiatt W.M., Patel S.G., O’Sullivan B., Brandwein M.S., Ridge J.A., Migliacci J.C., Loomis A.M., Shah J.P. (2017). Head and neck cancers-major changes in the American Joint Committee on cancer eighth edition cancer staging manual. CA Cancer J. Clin..

[80] Carrillo J., Carrillo L.C., Cano A., Ramirez–Ortega M.C., Chanona J.G., Avilés A., Herrera-Goepfert R., Corona-Rivera J., Ochoa-Carrillo F.J., Oñate-Ocaña L.F. (2016). Retrospective cohort study of prognostic factors in patients with oral cavity and oropharyngeal squamous cell carcinoma. Head Neck.

[81] Low T.-H., Gao K., Gupta R., Clifford A., Elliott M., Ch’Ng S., Milross C., Clark J.R. (2016). Factors predicting poor outcomes in T1N0 oral squamous cell carcinoma: Indicators for treatment intensification. ANZ J. Surg..

[82] Subramaniam N., Ms S.M., Balasubramanian D., Low T.-H., Vidhyadharan S., Clark J.R., Thankappan K., Iyer S. (2018). Adverse pathologic features in T1/2 oral squamous cell carcinoma classified by the American Joint Committee on Cancer eighth edition and implications for treatment. Head Neck.

[83] Lee L., De Paz D., Lin C., Fan K., Wang H., Hsieh C., Lee L., Yen T., Liao C., Yeh C. (2019). Prognostic impact of extratumoral perineural invasion in patients with oral cavity squamous cell carcinoma. Cancer Med..

[84] Caponio V.C.A., Troiano G., Togni L., Zhurakivska K., Santarelli A., Laino L., Rubini C., Muzio L.L., Mascitti M. (2021). Pattern and localization of perineural invasion predict poor survival in oral tongue carcinoma. Oral Dis..

[85] Chatzistefanou I., Lubek J., Markou K., Ord R.A. (2014). The role of neck dissection and postoperative adjuvant radiotherapy in cN0 patients with PNI-positive squamous cell carcinoma of the oral cavity. Oral Oncol..

[86] Nguyen E., McKenzie J., Clarke R., Lou S., Singh T. (2021). The Indications for Elective Neck Dissection in T1N0M0 Oral Cavity Squamous Cell Carcinoma. J. Oral Maxillofac. Surg..

[87] Feng Z., Cheng A., Alzahrani S., Li B., Han Z., Ward B.B. (2020). Elective Neck Dissection in T1N0M0 Oral Squamous Cell Carcinoma: When Is It Necessary?. J. Oral Maxillofac. Surg..

[88] Liao C.-T., Chang J.T.-C., Wang H.-M., Ng S.-H., Hsueh C., Lee L.-Y., Lin C.-H., Chen I.-H., Huang S.-F., Cheng A.-J. (2008). Does Adjuvant Radiation Therapy Improve Outcomes In pT1-3N0 Oral Cavity Cancer with Tumor-Free Margins and Perineural Invasion?. Int. J. Radiat. Oncol..

[89] Nair D., Mair M., Singhvi H., Mishra A., Nair S.V., Agrawal J., Chaturvedi P. (2018). Perineural invasion: Independent prognostic factor in oral cancer that warrants adjuvant treatment. Head Neck.

[90] Rajappa S., Ram D., Shukla H., Mandal G., Venkatasubramaniyan M., Dubey A., Agarwal M., Kumar R., Dewan A. (2019). Oncological benefits of postoperative radiotherapy in node-negative early stage cancer of the oral cavity with isolated perineural invasion. Br. J. Oral Maxillofac. Surg..

[91] Chinn S., Spector M.E., Bellile E.L., McHugh J.B., Gernon T.J., Bradford C.R., Wolf G.T., Eisbruch A., Chepeha D.B. (2013). Impact of Perineural Invasion in the Pathologically N0 Neck in Oral Cavity Squamous Cell Carcinoma. Otolaryngol. Neck Surg..

[92] Fan K.-H., Chen Y.-C., Lin C.-Y., Kang C.-J., Lee L.-Y., Huang S.-F., Liao C.-T., Ng S.-H., Wang H.-M., Chang J.T.-C. (2017). Postoperative radiotherapy with or without concurrent chemotherapy for oral squamous cell carcinoma in patients with three or more minor risk factors: A propensity score matching analysis. Radiat. Oncol..

[93] Babar A., Woody N., Ghanem A., Tsai J., Dunlap N., Schymick M., Liu H., Burkey B., Lamarre E., Ku J. (2021). Outcomes of Post-Operative Treatment with Concurrent Chemoradiotherapy (CRT) in High-Risk Resected Oral Cavity Squamous Cell Carcinoma (OCSCC): A Multi-Institutional Collaboration. Curr. Oncol..

[94] Scanlon C.S., Banerjee R., Inglehart R.C., Liu M., Russo N., Hariharan A., Van Tubergen E.A., Corson S.L., Asangani I., Mistretta C.M. (2015). Galanin modulates the neural niche to favour perineural invasion in head and neck cancer. Nat. Commun..

[95] Amit M., Takahashi H., Dragomir M.P., Lindemann A., Gleber-Netto F.O., Pickering C.R., Anfossi S., Osman A.A., Cai Y., Wang R. (2020). Loss of p53 drives neuron reprogramming in head and neck cancer. Nature.

[96] Kolokythas A., Cox D.P., Dekker N., Schmidt B.L. (2010). Nerve Growth Factor and Tyrosine Kinase A Receptor in Oral Squamous Cell Carcinoma: Is There an Association with Perineural Invasion?. J. Oral Maxillofac. Surg..

[97] Pascual G., Domínguez D., Elosúa-Bayes M., Beckedorff F., Laudanna C., Bigas C., Douillet D., Greco C., Symeonidi A., Hernández I. (2021). Dietary palmitic acid promotes a prometastatic memory via Schwann cells. Nature.

[98] Fujii-Nishimura Y., Yamazaki K., Masugi Y., Douguchi J., Kurebayashi Y., Kubota N., Ojima H., Kitago M., Shinoda M., Hashiguchi A. (2018). Mesenchymal-epithelial transition of pancreatic cancer cells at perineural invasion sites is induced by Schwann cells. Pathol. Int..

[99] Chen S.-H., Zhang B.-Y., Zhou B., Zhu C.-Z., Sun L.-Q., Feng Y.-J. (2019). Perineural invasion of cancer: A complex crosstalk between cells and molecules in the perineural niche. Am. J. Cancer Res..

[100] Amit M., Na’Ara S., Gil Z. (2016). Mechanisms of cancer dissemination along nerves. Nat. Rev. Cancer.

[101] Magnon C., Hall S.J., Lin J., Xue X., Gerber L., Freedland S.J., Frenette P.S. (2013). Autonomic Nerve Development Contributes to Prostate Cancer Progression. Science.

[102] Zahalka A.H., Arnal-Estapé A., Maryanovich M., Nakahara F., Cruz C.D., Finley L.W.S., Frenette P.S. (2017). Adrenergic nerves activate an angio-metabolic switch in prostate cancer. Science.

[103] Huang E.J., Reichardt L.F. (2001). Neurotrophins: Roles in Neuronal Development and Function. Annu. Rev. Neurosci..

[104] Demir I.E., Tieftrunk E., Schorn S., Friess H., Ceyhan G.O. (2016). Nerve growth factor & TrkA as novel therapeutic targets in cancer. Biochim. Biophys. Acta.

[105] Ma J., Jiang Y., Jiang Y., Sun Y., Zhao X. (2008). Expression of nerve growth factor and tyrosine kinase receptor A and correlation with perineural invasion in pancreatic cancer. J. Gastroenterol. Hepatol..

[106] Djakiew D., Pflug B.R., Delsite R., Onoda M., Lynch J.H., Arand G., Thompson E.W. (1993). Chemotaxis and chemokinesis of human prostate tumor cell lines in response to human prostate stromal cell secretory proteins containing a nerve growth factor-like protein. Cancer Res..

[107] Bapat A.A., Munoz R.M., Von Hoff D.D., Han H. (2016). Blocking Nerve Growth Factor Signaling Reduces the Neural Invasion Potential of Pancreatic Cancer Cells. PLoS ONE.

[108] Saloman J.L., Singhi A.D., Hartman D.J., Normolle D.P., Albers K.M., Davis B.M. (2018). Systemic Depletion of Nerve Growth Factor Inhibits Disease Progression in a Genetically Engineered Model of Pancreatic Ductal Adenocarcinoma. Pancreas.

[109] Shen W.-R., Wang Y.-P., Chang J.Y.-F., Yu S.-Y., Chen H.-M., Chiang C.-P. (2014). Perineural invasion and expression of nerve growth factor can predict the progression and prognosis of oral tongue squamous cell carcinoma. J. Oral Pathol. Med..

[110] Lin C., Ren Z., Yang X., Yang R., Chen Y., Liu Z., Dai Z., Zhang Y., He Y., Zhang C. (2020). Nerve growth factor (NGF)-TrkA axis in head and neck squamous cell carcinoma triggers EMT and confers resistance to the EGFR inhibitor erlotinib. Cancer Lett..

[111] Yu E.-H., Lui M.-T., Tu H.-F., Wu C.-H., Lo W.-L., Yang C.-C., Chang K.-W., Kao S.-Y. (2013). Oral carcinoma with perineural invasion has higher nerve growth factor expression and worse prognosis. Oral Dis..

[112] Alkhadar H., Macluskey M., White S., Ellis I. (2020). Nerve growth factor-induced migration in oral and salivary gland tumour cells utilises the PI3K/Akt signalling pathway: Is there a link to perineural invasion?. J. Oral Pathol. Med..

[113] Grille S.J., Bellacosa A., Upson J., Klein-Szanto A.J., Van Roy F., Lee-Kwon W., Donowitz M., Tsichlis P.N., LaRue L. (2003). The protein kinase Akt induces epithelial mesenchymal transition and promotes enhanced motility and invasiveness of squamous cell carcinoma lines. Cancer Res..

[114] Yang J., Antin P., Berx G., Blanpain C., Brabletz T., Bronner M., Campbell K., Cano A., Casanova J., Christofori G. (2020). Guidelines and definitions for research on epithelial–mesenchymal transition. Nat. Rev. Mol. Cell Biol..

[115] Gonzalez A., Moya-Alvarado G., Gonzalez-Billaut C., Bronfman F.C. (2016). Cellular and molecular mechanisms regulating neuronal growth by brain-derived neurotrophic factor. Cytoskeleton.

[116] Jia S., Wang W., Hu Z., Shan C., Wang L., Wu B., Yang Z., Yang X., Lei D. (2015). BDNF mediated TrkB activation contributes to the EMT progression and the poor prognosis in human salivary adenoid cystic carcinoma. Oral Oncol..

[117] Kowalski P.J., Paulino A.F. (2002). Perineural invasion in adenoid cystic carcinoma: Its causation/promotion by brain-derived neurotrophic factor. Hum. Pathol..

[118] Okugawa Y., Tanaka K., Inoue Y., Kawamura M., Kawamoto A., Hiro J., Saigusa S., Toiyama Y., Ohi M., Uchida K. (2013). Brain-derived neurotrophic factor/tropomyosin-related kinase B pathway in gastric cancer. Br. J. Cancer.

[119] Albini A. (1998). Tumor and endothelial cell invasion of basement membranes. The matrigel chemoinvasion assay as a tool for dissecting molecular mechanisms. Pathol. Oncol. Res..

[120] Miknyoczki S.J., Lang D., Huang L., Klein-Szanto A.J., Dionne C.A., Ruggeri B.A. (1999). Neurotrophins and Trk receptors in human pancreatic ductal adenocarcinoma: Expression patterns and effects onIn vitro invasive behavior. Int. J. Cancer.

[121] Ketterer K., Rao S., Friess H., Weiss J., Büchler M.W., Korc M. (2003). Reverse transcription-PCR analysis of laser-captured cells points to potential paracrine and autocrine actions of neurotrophins in pancreatic cancer. Clin. Cancer Res..

[122] Sclabas G.M., Fujioka S., Schmidt C., Li Z., A I Frederick W., Yang W., Yokoi K., Evans D.B., Abbruzzese J.L., Hess K.R. (2005). Overexpression of tropomysin-related kinase B in metastatic human pancreatic cancer cells. Clin. Cancer Res..

[123] Yilmaz T., Jiffar T., de la Garza G., Lin H., MacIntyre T., Brown J.L., Myers J.N., Kupferman M.E. (2010). Therapeutic targeting of Trk supresses tumor proliferation and enhances cisplatin activity in HNSCC. Cancer Biol. Ther..

[124] Zhu L., A Werner J., Mandic R. (2007). Implications of tropomyosin-related kinase B (TrkB) in head and neck cancer. Anticancer Res..

[125] E Kupferman M., Jiffar T., El-Naggar A., Yilmaz T., Zhou G., Xie T., Feng L., Wang J., Holsinger F.C., Yu D. (2010). TrkB induces EMT and has a key role in invasion of head and neck squamous cell carcinoma. Oncogene.

[126] Dudás J., Bitsche M., Schartinger V., Falkeis C., Sprinzl G.M., Riechelmann H. (2011). Fibroblasts produce brain-derived neurotrophic factor and induce mesenchymal transition of oral tumor cells. Oral Oncol..

[127] Jing S., Wen D., Yu Y., Holst P.L., Luo Y., Fang M., Tamir R., Antonio L., Hu Z., Cupples R. (1996). GDNF–Induced Activation of the Ret Protein Tyrosine Kinase Is Mediated by GDNFR-α, a Novel Receptor for GDNF. Cell.

[128] He S., Chen C.-H., Chernichenko N., Bakst R.L., Barajas F., Deborde S., Allen P.J., Vakiani E., Yu Z., Wong R.J. (2014). GFR 1 released by nerves enhances cancer cell perineural invasion through GDNF-RET signaling. Proc. Natl. Acad. Sci. USA.

[129] Gil Z., Cavel O., Kelly K., Brader P., Rein A., Gao S.P., Carlson D.L., Shah J., Fong Y., Wong R.J. (2010). Paracrine Regulation of Pancreatic Cancer Cell Invasion by Peripheral Nerves. J. Natl. Cancer Inst..

[130] Ban K., Feng S., Shao L., Ittmann M. (2017). RET Signaling in Prostate Cancer. Clin. Cancer Res..

[131] Chuang J.-Y., Tsai C.-F., Chang S.-W., Chiang I.-P., Huang S.-M., Lin H.-Y., Yeh W.-L., Lu D.-Y. (2013). Glial cell line-derived neurotrophic factor induces cell migration in human oral squamous cell carcinoma. Oral Oncol..

[132] Lin C., Cao W., Ren Z., Tang Y., Zhang C., Yang R., Chen Y., Liu Z., Peng C., Wang L. (2017). GDNF secreted by nerves enhances PD-L1 expression via JAK2-STAT1 signaling activation in HNSCC. Oncoimmunology.

[133] Elliott-Hunt C.R., Pope R.J.P., Vanderplank P., Wynick D. (2007). Activation of the galanin receptor 2 (GalR2) protects the hippocampus from neuronal damage. J. Neurochem..

[134] Berger A., Lang R., Moritz K., Santic R., Hermann A., Sperl W., Kofler B. (2004). Galanin Receptor Subtype GalR2 Mediates Apoptosis in SH-SY5Y Neuroblastoma Cells. Endocrinology.

[135] Gilaberte Y., Vera J., Coscojuela C., Roca M., Parrado C., González S. (2007). Expression of Galanin in Melanocytic Tumors. Actas Dermosifiliogr..

[136] Kim K.Y., Kee M.K., Chong S.A., Nam M.J. (2007). Galanin Is Up-Regulated in Colon Adenocarcinoma. Cancer Epidemiol. Biomark. Prev..

[137] Rauch I., Kofler B. (2010). The Galanin System in Cancer. Exp. Suppl..

[138] Berger A., Santic R., Hauser-Kronberger C., Schilling F.H., Kogner P., Ratschek M., Gamper A., Jones N., Sperl W., Kofler B. (2005). Galanin and galanin receptors in human cancers. Neuropeptides.

[139] Xia C.-Y., Yuan C.-X., Yuan C.-G. (2005). Galanin inhibits the proliferation of glial olfactory ensheathing cells. Neuropeptides.

[140] Pearlstein R.P., Benninger M.S., Carey T.E., Zarbo R.J., Torres F.X., Rybicki B.A., Van Dyke D.L. (1998). Loss of 18q predicts poor survival of patients with squamous cell carcinoma of the head and neck. Genes Chromosom. Cancer.

[141] Takebayashi S., Ogawa T., Jung K.Y., Muallem A., Mineta H., Fisher S.G., Grenman R., Carey T. (2000). Identification of new minimally lost regions on 18q in head and neck squamous cell carcinoma. Cancer Res..

[142] Jacoby A.S., Webb G.C., Liu M.L., Kofler B., Hort Y.J., Fathi Z., Bottema C., Shine J., Iismaa T.P. (1997). Structural Organization of the Mouse and Human GALR1 Galanin Receptor Genes (GalnrandGALNR) and Chromosomal Localization of the Mouse Gene. Genomics.

[143] Henson B.S., Neubig R.R., Jang I., Ogawa T., Zhang Z., Carey T., D’Silva N.J. (2005). Galanin Receptor 1 Has Anti-proliferative Effects in Oral Squamous Cell Carcinoma. J. Biol. Chem..

[144] Banerjee R., Henson B.S., Russo N., Tsodikov A., D’Silva N.J. (2011). Rap1 mediates galanin receptor 2-induced proliferation and survival in squamous cell carcinoma. Cell. Signal..

[145] Kanazawa T., Iwashita T., Kommareddi P., Nair T., Misawa K., Misawa Y., Ueda Y., Tono T., E Carey T. (2007). Galanin and galanin receptor type 1 suppress proliferation in squamous carcinoma cells: Activation of the extracellular signal regulated kinase pathway and induction of cyclin-dependent kinase inhibitors. Oncogene.

[146] Misawa K., Kanazawa T., Misawa Y., Uehara T., Imai A., Takahashi G., Takebayashi S., Cole A., E Carey T., Mineta H. (2013). Galanin Has Tumor Suppressor Activity and Is Frequently Inactivated by Aberrant Promoter Methylation in Head and Neck Cancer. Transl. Oncol..

[147] Kanazawa T., Kommareddi P.K., Iwashita T., Kumar B., Misawa K., Misawa Y., Jang I., Nair T.S., Iino Y., Carey T. (2009). Galanin Receptor Subtype 2 Suppresses Cell Proliferation and Induces Apoptosis in p53 Mutant Head and Neck Cancer Cells. Clin. Cancer Res..

[148] Williams E.J., Furness J., Walsh F.S., Doherty P. (1994). Activation of the FGF receptor underlies neurite outgrowth stimulated by L1, N-CAM, and N-cadherin. Neuron.

[149] Hinsby A.M., Berezin V., Bock E. (2004). Molecular mechanisms of NCAM function. Front. Biosci..

[150] Brugière C., El Bouchtaoui M., Leboeuf C., Gapihan G., El Far R.A., Sy M., Lepage A.L., Ratajczak P., Janin A., Verneuil L. (2018). Perineural Invasion in Human Cutaneous Squamous Cell Carcinoma Is Linked to Neurotrophins, Epithelial-Mesenchymal Transition, and NCAM1. J. Investig. Dermatol..

[151] Deborde S., Omelchenko T., Lyubchik A., Zhou Y., He S., McNamara W.F., Chernichenko N., Lee S.-Y., Barajas F., Chen C.-H. (2016). Schwann cells induce cancer cell dispersion and invasion. J. Clin. Investig..

[152] Li R. (2003). Neural cell adhesion molecule is upregulated in nerves with prostate cancer invasion. Hum. Pathol..

[153] Stierli S., Imperatore V., Lloyd A.C. (2019). Schwann cell plasticity-roles in tissue homeostasis, regeneration, and disease. Glia.

[154] Vural E., Hutcheson J., Korourian S., Kechelava S., Hanna E. (2000). Correlation of Neural Cell Adhesion Molecules with Perineural Spread of Squamous Cell Carcinoma of the Head and Neck. Otolaryngol. Head Neck Surg..

[155] McLaughlin R.B., Montone K.T., Wall S.J., Chalian A.A., Weinstein G.S., Roberts S.A., Wolf P.F., Weber R.S. (1999). Nerve cell adhesion molecule expression in squamous cell carcinoma of the head and neck: A predictor of propensity toward perineural spread. Laryngoscope.

[156] Solares C.A., Brown I., Boyle G.M., Parsons P.G., Panizza B. (2009). Neural cell adhesion molecule expression: No correlation with perineural invasion in cutaneous squamous cell carcinoma of the head and neck. Head Neck.

[157] Banh R.S., Biancur D.E., Yamamoto K., Sohn A.S., Walters B., Kuljanin M., Gikandi A., Wang H., Mancias J.D., Schneider R.J. (2020). Neurons Release Serine to Support mRNA Translation in Pancreatic Cancer. Cell.

[158] Zeng Q., Michael I.P., Zhang P., Saghafinia S., Knott G., Jiao W., McCabe B.D., Galván J.A., Robinson H.P.C., Zlobec I. (2019). Synaptic proximity enables NMDAR signalling to promote brain metastasis. Nature.

[159] Mauffrey P., Tchitchek N., Barroca V., Bemelmans A.-P., Firlej V., Allory Y., Roméo P.-H., Magnon C. (2019). Progenitors from the central nervous system drive neurogenesis in cancer. Nature.

[160] Bakst R.L., Lee N., He S., Chernichenko N., Chen C.-H., Linkov G., Le H.C., Koutcher J., Vakiani E., Wong R.J. (2012). Radiation Impairs Perineural Invasion by Modulating the Nerve Microenvironment. PLoS ONE.

[161] Meldolesi J. (2018). Neurotrophin Trk Receptors: New Targets for Cancer Therapy. Rev. Physiol. Biochem. Pharmacol..

